# Supplementary material for: Hostility has a trivial effect on persuasiveness of rebutting science denialism on social media
Source: Commun Psychol. 2023 Dec 11;1:39. doi: 10.1038/s44271-023-00041-w (PMC11332242; doi:10.1038/s44271-023-00041-w)
Supplement: Supplementary file 1 — Supplementary Information [file 44271_2023_41_MOESM1_ESM.pdf]

Supplementary Information

Hostility has a trivial effect on persuasiveness of rebutting science denialism on social media

Philipp Schmid[1,2,3], Benedikt Werner[4]

[1]Institute for Planetary Health Behaviour, University of Erfurt, Erfurt, Germany
[2]Centre for Language Studies, Radboud University, Nijmegen, The Netherlands
[3]Health Communication, Department of Implementation Research, Bernhard-Nocht-Institute for Tropical Medicine, Hamburg, Germany
[4]Department for Applied Microeconomics, University of Erfurt, Erfurt, Germany

Table of Contents

SUPPLEMENTARY TABLE 1. SPECIFIC DEMOGRAPHICS OF EACH CONDITION IN EXPERIMENT 1. 5

SUPPLEMENTARY TABLE 2. SPECIFIC DEMOGRAPHICS OF EACH CONDITION IN EXPERIMENT 2. 6

SUPPLEMENTARY TABLE 3. SPECIFIC DEMOGRAPHICS OF EACH CONDITION IN EXPERIMENT 3. 7

SUPPLEMENTARY TABLE 4. SPECIFIC DEMOGRAPHICS OF EACH CONDITION IN EXPERIMENT 4. 8

SUPPLEMENTARY TABLE 5. OVERVIEW OF MEASURES USED IN EXPERIMENTS 1-4. 11

SUPPLEMENTARY TABLE 6: ASSUMPTION TESTS FOR GLM INCLUDING ANCOVA. 12

SUPPLEMENTARY TABLE 7. ANCOVA RESULTS OF EXPERIMENT 1: IMPACT OF HOSTILITY (ADVOCATE AND DENIER) ON INTENTION AND ATTITUDE CONTROLLED FOR BASELINE VALUES. 13

SUPPLEMENTARY TABLE 8. MODERATED MEDIATOR ANALYSES EXPERIMENT 1: IMPACT OF HOSTILITY (ADVOCATE) ON INDIRECT EFFECTS OF HOMOGENEITY OF CONDITIONS ON INTENTION (MODEL A) AND ATTITUDE (MODEL B) VIA EXPECTANCY RATINGS. 14

SUPPLEMENTARY TABLE 9. MEDIATOR ANALYSES EXPERIMENT 1: IMPACT OF HOSTILITY (ADVOCATE) ON ATTITUDE (MODEL 1) AND INTENTION (MODEL 2) VIA COMPETENCE RATINGS. 15

SUPPLEMENTARY TABLE 10. MEDIATOR ANALYSES EXPERIMENT 1: IMPACT OF HOSTILITY (DENIER) ON ATTITUDE (MODEL 3) AND INTENTION (MODEL 4) VIA COMPETENCE RATINGS. 16

SUPPLEMENTARY TABLE 11. MODERATOR ANALYSES EXPERIMENT 1: IMPACT OF HOSTILITY (ADVOCATE) ON INTENTION (MODEL 5) AND ATTITUDE (MODEL 6) AS A FUNCTION OF SOCIAL MEDIA USE. 17

SUPPLEMENTARY TABLE 12. MODERATOR ANALYSES EXPERIMENT 1: IMPACT OF HOSTILITY (DENIER) ON INTENTION (MODEL 7) AND ATTITUDE (MODEL 8) AS A FUNCTION OF SOCIAL MEDIA USE. 18

SUPPLEMENTARY TABLE 13. MODERATOR ANALYSES EXPERIMENT 1: IMPACT OF HOSTILITY (ADVOCATE) ON INTENTION (MODEL 9) AND ATTITUDE (MODEL 10) AS A FUNCTION OF VERBAL AGGRESSION. 19

SUPPLEMENTARY TABLE 14. MODERATOR ANALYSES EXPERIMENT 1: IMPACT OF HOSTILITY (DENIER) ON INTENTION (MODEL 11) AND ATTITUDE (MODEL 12) AS A FUNCTION OF VERBAL AGGRESSION. 20

SUPPLEMENTARY TABLE 15. ANCOVA RESULTS OF EXPERIMENT 2: IMPACT OF HOSTILITY (ADVOCATE AND DENIER) ON INTENTION AND ATTITUDE CONTROLLED FOR BASELINE VALUES. 21

SUPPLEMENTARY TABLE 16. MEDIATOR ANALYSES EXPERIMENT 2: IMPACT OF HOSTILITY (DENIER) ON ATTITUDE (MODEL 1) AND INTENTION (MODEL 2) VIA COMPETENCE RATINGS. 22

SUPPLEMENTARY TABLE 17. MODERATOR ANALYSES EXPERIMENT 2: IMPACT OF HOSTILITY (DENIER) ON INTENTION (MODEL 3) AND ATTITUDE (MODEL 4) AS A FUNCTION OF SOCIAL MEDIA USE. 23

SUPPLEMENTARY TABLE 18. MODERATOR ANALYSES EXPERIMENT 2: IMPACT OF HOSTILITY (DENIER) ON INTENTION (MODEL 5) AND ATTITUDE (MODEL 6) AS A FUNCTION OF VERBAL AGGRESSION. 24

SUPPLEMENTARY TABLE 19. ANCOVA RESULTS OF EXPERIMENT 3: IMPACT OF HOSTILITY (ADVOCATE AND DENIER) ON INTENTION AND ATTITUDE CONTROLLED FOR BASELINE VALUES. 25

SUPPLEMENTARY TABLE 20. MEDIATOR ANALYSES EXPERIMENT 3: IMPACT OF HOSTILITY (ADVOCATE) ON ATTITUDE (MODEL 1) AND INTENTION (MODEL 2) VIA COMPETENCE RATINGS. 26

SUPPLEMENTARY TABLE 21. MEDIATOR ANALYSES EXPERIMENT 3: IMPACT OF HOSTILITY (DENIER) ON ATTITUDE (MODEL 3) AND INTENTION (MODEL 4) VIA COMPETENCE RATINGS. 27

SUPPLEMENTARY TABLE 22. MODERATOR ANALYSES EXPERIMENT 3: IMPACT OF HOSTILITY (ADVOCATE) ON INTENTION (MODEL 5) AND ATTITUDE (MODEL 6) AS A FUNCTION OF VERBAL AGGRESSION. 28

SUPPLEMENTARY TABLE 23. MODERATOR ANALYSES EXPERIMENT 3: IMPACT OF HOSTILITY (DENIER) ON INTENTION (MODEL 7) AND ATTITUDE (MODEL 8) AS A FUNCTION OF VERBAL AGGRESSION. 29

SUPPLEMENTARY TABLE 24. MODERATOR ANALYSES EXPERIMENT 3: IMPACT OF HOSTILITY (ADVOCATE) ON INTENTION (MODEL 9) AND ATTITUDE (MODEL 10) AS A FUNCTION OF NEED FOR COGNITION. 30

SUPPLEMENTARY TABLE 25. MODERATOR ANALYSES EXPERIMENT 3: IMPACT OF HOSTILITY (DENIER) ON INTENTION (MODEL 11) AND ATTITUDE (MODEL 12) AS A FUNCTION OF NEED FOR COGNITION. 31

SUPPLEMENTARY TABLE 26. MODERATOR ANALYSES EXPERIMENT 3: IMPACT OF HOSTILITY (ADVOCATE) ON INTENTION (MODEL 13) AND ATTITUDE (MODEL 14) AS A FUNCTION OF ISSUE INVOLVEMENT. 32

SUPPLEMENTARY TABLE 27. MODERATOR ANALYSES EXPERIMENT 3: IMPACT OF HOSTILITY (DENIER) ON INTENTION (MODEL 15) AND ATTITUDE (MODEL 16) AS A FUNCTION OF ISSUE INVOLVEMENT. 33

SUPPLEMENTARY TABLE 28: ANCOVA RESULTS OF EXPERIMENT 4: IMPACT OF HOSTILITY (ADVOCATE AND DENIER) ON INTENTION AND ATTITUDE CONTROLLED FOR BASELINE VALUES. 34

SUPPLEMENTARY TABLE 29. MEDIATOR ANALYSES EXPERIMENT 4: IMPACT OF HOSTILITY (ADVOCATE) ON ATTITUDE (MODEL 1) AND INTENTION (MODEL 2) VIA COMPETENCE RATINGS. 35

SUPPLEMENTARY TABLE 30. MEDIATOR ANALYSES EXPERIMENT 4: IMPACT OF HOSTILITY (DENIER) ON ATTITUDE (MODEL 3) AND INTENTION (MODEL 4) VIA COMPETENCE RATINGS. 36

SUPPLEMENTARY TABLE 31. MODERATOR ANALYSES EXPERIMENT 4: IMPACT OF HOSTILITY (ADVOCATE) ON INTENTION (MODEL 5) AND ATTITUDE (MODEL 6) AS A FUNCTION OF VERBAL AGGRESSION. 37

SUPPLEMENTARY TABLE 32. MODERATOR ANALYSES EXPERIMENT 4: IMPACT OF HOSTILITY (DENIER) ON INTENTION (MODEL 7) AND ATTITUDE (MODEL 8) AS A FUNCTION OF VERBAL AGGRESSION. 38

SUPPLEMENTARY TABLE 33. MODERATOR ANALYSES EXPERIMENT 4: IMPACT OF HOSTILITY (ADVOCATE) ON INTENTION (MODEL 9) AND ATTITUDE (MODEL 10) AS A FUNCTION OF NEED FOR COGNITION. 39

SUPPLEMENTARY TABLE 34. MODERATOR ANALYSES EXPERIMENT 4: IMPACT OF HOSTILITY (DENIER) ON INTENTION (MODEL 11) AND ATTITUDE (MODEL 12) AS A FUNCTION OF NEED FOR COGNITION. 40

SUPPLEMENTARY TABLE 35. MODERATOR ANALYSES EXPERIMENT 4: IMPACT OF HOSTILITY (ADVOCATE) ON INTENTION (MODEL 13) AND ATTITUDE (MODEL 14) AS A FUNCTION OF ISSUE INVOLVEMENT. 41

SUPPLEMENTARY TABLE 36. MODERATOR ANALYSES EXPERIMENT 4: IMPACT OF HOSTILITY (DENIER) ON INTENTION (MODEL 15) AND ATTITUDE (MODEL 16) AS A FUNCTION OF ISSUE INVOLVEMENT. 42

SUPPLEMENTARY TABLE 37. IMPACT OF HOSTILITY (DENIER AND ADVOCATE) ON PERCEIVED AUTHENTICITY. 43

SUPPLEMENTARY TABLE 38. ROBUSTNESS CHECK OF EXPERIMENT 3: IMPACT OF HOSTILITY (ADVOCATE AND DENIER) ON INTENTION AND ATTITUDE AND COMPETENCE WITHOUT INDIVIDUALS THAT RESPOND IN HIGHLY SOCIAL DESIRABLE MANNER. 44

SUPPLEMENTARY TABLE 39. ROBUSTNESS CHECK OF EXPERIMENT 4: IMPACT OF HOSTILITY (ADVOCATE AND DENIER) ON INTENTION AND ATTITUDE AND COMPETENCE WITHOUT INDIVIDUALS THAT RESPOND IN HIGHLY SOCIAL DESIRABLE MANNER. 45

SUPPLEMENTARY TABLE 40. ROBUSTNESS CHECK OF EXPERIMENT 1: IMPACT OF HOSTILITY (ADVOCATE AND DENIER) ON INTENTION AND ATTITUDE AND COMPETENCE WITHOUT SPEEDERS. 46

SUPPLEMENTARY TABLE 41. ROBUSTNESS CHECK OF EXPERIMENT 2: IMPACT OF HOSTILITY (ADVOCATE AND DENIER) ON INTENTION AND ATTITUDE AND COMPETENCE WITHOUT SPEEDERS. 46

SUPPLEMENTARY TABLE 42. ROBUSTNESS CHECK OF EXPERIMENT 3: IMPACT OF HOSTILITY (ADVOCATE AND DENIER) ON INTENTION AND ATTITUDE AND COMPETENCE WITHOUT SPEEDERS. 47

SUPPLEMENTARY TABLE 43. ROBUSTNESS CHECK OF EXPERIMENT 4: IMPACT OF HOSTILITY (ADVOCATE AND DENIER) ON INTENTION AND ATTITUDE AND COMPETENCE WITHOUT SPEEDERS. 48

SUPPLEMENTARY TABLE 44. ROBUSTNESS CHECK OF EXPERIMENT 1: IMPACT OF HOSTILITY (ADVOCATE AND DENIER) ON INTENTION AND ATTITUDE AND COMPETENCE WITHOUT INATTENTIVE INDIVIDUALS. 49

SUPPLEMENTARY TABLE 45. ROBUSTNESS CHECK OF EXPERIMENT 2: IMPACT OF HOSTILITY (ADVOCATE AND DENIER) ON INTENTION AND ATTITUDE AND COMPETENCE WITHOUT INATTENTIVE INDIVIDUALS. 49

SUPPLEMENTARY TABLE 46. ROBUSTNESS CHECK OF EXPERIMENT 3: IMPACT OF HOSTILITY (ADVOCATE AND DENIER) ON INTENTION AND ATTITUDE AND COMPETENCE WITHOUT INATTENTIVE INDIVIDUALS. 50

SUPPLEMENTARY TABLE 47. ROBUSTNESS CHECK OF EXPERIMENT 4: IMPACT OF HOSTILITY (ADVOCATE AND DENIER) ON INTENTION AND ATTITUDE AND COMPETENCE WITHOUT INATTENTIVE INDIVIDUALS. 51

SUPPLEMENTARY REFERENCES 52

	Experiment 1 overall (*N* = 521)	Condition: Neutral denier/ Neutral advocate (*n* = 134)	Condition: Neutral denier/ Hostile advocate (*n* = 127)	Condition: Hostile denier/ Neutral advocate (*n* = 131)	Condition: Hostile denier/ Hostile advocate (*n* = 129)
Age Mean(Standard Deviation)	34.05 (10.46)	32.50 (9.60)	34.60 (9.76)	34.77 (11.21)	34.39 (11.12)
Gender					
Men	51.6% (269)	50.7% (68)	52.8% (67)	49.6% (65)	53.5% (69)
Women	47.6% (248)	49.3% (66)	45.7% (58)	49.6% (65)	45.7% (59)
Non-binary	0.6% (3)	-	1.6% (2)	-	0.8% (1)
Education					
Low	-	-	-	-	-
Middle	96.7% (504)	97.2% (132)	96.9% (123)	95.4% (125)	97.7% (126)
High	2.7% (14)	1.5% (2)	3.1% (4)	3.8% (5)	2.3% (3)
Attitude *M*(*SD*)					
Baseline (Pre)	87.30 (19.62)	87.31 (16.17)	85.39 (23.07)	90.12 (19.26)	86.30 (19.45)
Post measure	82.23 (22.52)	83.79 (18.52)	80.40 (25.05)	86.13 (21.10)	78.73 (24.50)
Intention *M*(*SD*)					
Baseline (Pre)	84.13 (23.16)	84.70 (21.05)	83.60 (25.63)	86.51 (22.28)	81.65 (23.56)
Post measure	79.11 (26.20)	81.22 (22.24)	77.17 (28.70)	82.82 (25.06)	75.06 (28.04)
Perceived Competence *M*(*SD*)					
Denier	41.36 (33.59)	51.87 (30.57)	57.74 (32.18)	27.10 (32.05)	28.81 (28.32)
Advocate	70.41 (29.33)	78.98 (23.21)	55.90 (31.11)	87.02 (19.97)	58.91 (29.61)
Expectancy advocate *M*(*SD*)	68.78 (24.37)	73.88 (21.04)	65.16 (28.65)	70.36 (21.93)	65.44 (24.54)
Verbal aggression *M*(*SD*)	54.59 (23.59)	55.89 (24.36)	55.56 (23.22)	53.69 (24.36)	53.19 (22.48)
Frequency social media use	76.01 (27.31)	76.50 (25.79)	74.67 (27.37)	76.08 (29.32)	76.74 (26.96)

Supplementary Table 1. Specific demographics of each condition in Experiment 1.
To be a participant on Prolific, you must be *over* 18 years of age. Thus, responses below 18 ($n = 1$) were treated as implausible and were not included in the calculation of the sample mean.

	**Experiment 2 overall (*N* = 310)**	**Condition: Neutral denier / Hostile advocate (*n* = 76)**	**Condition: Neutral denier / Advocate absent (*n* = 81)**	**Condition: Hostile denier / Hostile advocate (*n* = 75)**	**Condition: Hostile denier / Advocate absent (*n* = 78)**
Age Mean(Standard Deviation)	34.46 (11.25)	34.62 (11.88)	33.02 (9.16)	35.51 (12.42)	34.79 (11.50)
Gender					
Men	47.4% (147)	48.7% (37)	45.7% (37)	45.3% (34)	50% (39)
Women	50% (155)	48.7% (37)	50.6% (41)	52% (39)	48.7% (38)
Non-binary	2.6% (8)	2.6% (2)	3.7% (3)	2.7% (2)	1.3% (1)
Education					
Low	-	-	-	-	-
Middle	94.2% (292)	94.7% (72)	100% (81)	92%% (69)	89.7% (70)
High	5.5% (17)	3.9% (3)	-	8% (6)	10.3% (8)
Attitude *M*(*SD*)					
Baseline (Pre)	89.19 (16.27)	89.84 (17.65)	89.85 (14.77)	88.22 (17.50)	88.82 (15.37)
Post measure	82.78 (21.97)	85.31 (21.33)	80.59 (22.05)	84.52 (19.62)	80.91 (24.51)
Intention *M*(*SD*)					
Baseline (Pre)	86.77 (20.26)	87.06 (21.53)	89.09 (15.18)	85.56 (21.63)	85.26 (22.31)
Post measure	81.56 (23.75)	81.36 (26.10)	80.25 (24.88)	83.78 (21.57)	80.98 (22.43)
Perceived Competence *M*(*SD*)					
Denier	34.78 (31.17)	50.00 (30.31)	49.18 (29.45)	20.89 (25.27)	18.38 (24.11)
Verbal aggression *M*(*SD*)	51.51 (23.17)	54.09 (19.40)	47.74 (25.58)	48.74 (24.69)	55.56 (21.84)
Frequency social media use	76.88 (27.41)	76.75 (28.03)	77.98 (27.24)	75.78 (28.25)	76.92 (26.62)

Supplementary Table 2. Specific demographics of each condition in Experiment 2.

	**Experiment 3 overall (*N* = 1200)**	**Condition: Neutral denier / Neutral advocate (*n* = 195)**	**Condition: Neutral denier / Hostile advocate (*n* = 197)**	**Condition: Neutral denier / Advocate absent (*n* = 201)**	**Condition: Hostile denier / Neutral advocate (*n* = 204)**	**Condition: Hostile denier / Hostile advocate (*n* = 204)**	**Condition: Hostile denier / Advocate absent (*n* = 199)**
Age Mean(Standard Deviation)	33.00 (12.17)	33.70 (13.07)	31.71 (10.61)	33.80 (13.56)	33.94 (12.24)	32.17 (11.52)	32.64 (11.76)
Gender							
Men	48.8% (585)	49.7% (97)	44.2% (87)	57.2% (115)	49.5% (101)	43.6% (89)	48.2% (96)
Women	48.8% (585)	47.2% (92)	52.8% (104)	40.8% (82)	49% (100)	53.4% (109)	49.2% (98)
Non-binary	2.3% (28)	2.6% (5)	2.5% (5)	2% (4)	1.5% (3)	2.9% (6)	2.5% (5)
Education							
Low	0.6% (7)	-	1% (2)	1% (2)	-	1.5% (3)	-
Middle	95.1% (1141)	96.9% (189)	94.9% (187)	96.5% (194)	96.1% (196)	90.7% (185)	95.5% (190)
High	3.9% (47)	2.6% (5)	3.6% (7)	2.5% (5)	3.9% (8)	7.8% (16)	3% (6)
Attitude *M*(*SD*)							
Baseline (Pre)	85.47 (18.94)	83.97 (21.37)	86.17 (18.82)	85.76 (18.75)	86.42 (17.54)	85.52 (18.00)	84.90 (19.18)
Post measure	80.76 (22.71)	81.51 (23.46)	81.28 (22.80)	73.57 (25.85)	85.19 (19.12)	83.48 (18.47)	79.41 (24.21)
Intention *M*(*SD*)							
Baseline (Pre)	85.53 (22.68)	83.67 (25.15)	85.70 (24.51)	85.90 (22.37)	85.70 (22.10)	87.01 (19.75)	85.09 (22.12)
Post measure	80.33 (27.04)	80.51 (27.32)	81.64 (27.30)	73.96 (29.61)	83.25 (25.60)	83.42 (22.95)	79.15 (28.26)
Perceived Competence *M*(*SD*)	--						
Denier	35.76 (31.89)	43.08 (31.78)	50.17 (30.77)	47.84 (28.50)	29.41 (34.91)	25.33 (27.26)	19.35 (23.83)
Advocate	64.31 (32.24)	73.42 (27.99)	55.08 (31.04)	--	75.08 (33.48)	53.76 (30.00)	
Expectancy advocate *M*(*SD*)	--	--	. --	--	24.68 (18.29)		
Verbal aggression *M*(*SD*)	24.13 (18.69)	23.55 (18.55)	23.10 (18.21)	22.99 (18.34)	24.68 (18.29)	26.35 (19.83)	24.06 (18.85)
Need for Cognition *M*(*SD*)	65.39 (18.90)	66.79 (18.93)	64.87 (24.17)	66.58 (18.70)	65.48 (18.84)	64.75 (18.95)	63.86 (20.01)
Issue Involvement *M*(*SD*)	59.90 (24.90)	59.44 (25.90)	59.94 (24.17)	60.32 (24.64)	60.05 (25.07)	59.35 (24.73)	60.22 (25.19)

Supplementary Table 3. Specific demographics of each condition in Experiment 3.

	**Experiment 4 overall (*N* = 1195)**	**Condition: Neutral denier / Neutral advocate (*n* = 202)**	**Condition: Neutral denier / Hostile advocate (*n* = 198)**	**Condition: Neutral denier / Advocate absent (*n* = 200)**	**Condition: Hostile denier / Neutral advocate (*n* = 198)**	**Condition: Hostile denier / Hostile advocate (*n* = 197)**	**Condition: Hostile denier / Advocate absent (*n* = 200)**
Age Mean(Standard Deviation)	33.71 (12.12)	34.28 (12.54)	34.79 (12.25)	32.97 (11.11)	33.84 (12.80)	33.28 (12.83)	33.09 (11.09)
Gender							
Men	51% (610)	51.5% (104)	48% (95)	56.5% (113)	53% (105)	45.7% (90)	51.5% (103)
Women	46.7% (558)	47% (95)	49% (97)	41% (82)	44.9% (89)	52.3% (103)	46% (92)
Non-binary	2.1% (25)	1.5% (3)	3% (6)	2.5% (5)	2% (4)	2% (4)	1.5% (3)
Education							
Low	0.7% (8)	0.5% (1)	1.5% (3)	0.5% (1)	1% (2)	0.5% (1)	-
Middle	94.6% (1131)	95% (192)	93.4% (185)	95.5% (190)	94.4% (187)	95.9% (189)	94% (188)
High	4.3% (51)	4.5% (9)	4.4% (9)	4% (8)	4% (8)	3% (6)	5.5% (11)
Attitude *M*(*SD*)							
Baseline (Pre)	60.01 (26.35)	58.52 (25.99)	60.00 (25.58)	60.96 (26.97)	58.48 (28.22)	61.53 (25.90)	60.58 (25.52)
Post measure	58.41 (28.03)	61.49 (26.68)	59.97 (25.95)	52.48 (29.02)	60.50 (29.12)	62.23 (27.14)	53.83 (28.88)
Intention *M*(*SD*)							
Baseline (Pre)	51.73 (31.26)	49.51 (32.06)	50.08 (29.64)	54.42 (31.32)	51.60 (32.20)	51.69 (30.95)	53.08 (31.44)
Post measure	52.71 (31.71)	53.55 (31.63)	52.86 (29.19)	47.50 (34.07)	55.22 (31.74)	56.68 (30.52)	50.50 (32.40)
Perceived Competence *M*(*SD*)							
Denier	45.45 (32.37)	57.43 (31.63)	61.03 (28.11)	65.42 (26.31)	32.74 (33.01)	28.43 (27.28)	27.33 (27.46)
Advocate	62.20 (30.38)	71.86 (22.47)	49.83 (29.80)	--	75.08 (30.45)	51.78 (29.47)	--
Expectancy advocate *M*(*SD*)	--		--	--			--
Verbal aggression *M*(*SD*)	22.24 (18.34)	21.25 (17.80)	24.14 (18.96)	23.16 (18.88)	21.51 (18.22)	22.42 (18.83)	20.99 (17.33)
Need for Cognition *M*(*SD*)	63.48 (20.73)	61.90 (21.95)	64.42 (18.82)	64.23 (20.90)	62.82 (19.28)	63.20 (21.94)	64.33 (21.35)
Issue Involvement *M*(*SD*)	45.40 (25.06)	43.73 (22.76)	48.86 (25.13)	40.88 (24.79)	45.37 (27.16)	48.22 (24.91)	45.42 (24.88)

Supplementary Table 4. Specific demographics of each condition in Experiment 4.

concept	scale type and reliability*	wording	source of adapted items
**primary outcomes**			
intention to get vaccinated (Exp. 1; Exp. 2; Exp. 3)	7-point rating scale	If you had the opportunity to get vaccinated against dysomeria, what would you do?<br>(1 = I will definitely not get vaccinated, 7 = I will definitely get vaccinated)	[1,2]
attitude towards vaccination (Exp. 1; Exp. 2; Exp. 3)	mean score of 7-point rating scales (Cronbach's $\alpha1_{pre}$ = .92; $\alpha1_{post}$ = .95; $\alpha2_{pre}$ = .91; $\alpha2_{post}$ = .95; $\alpha3_{pre}$ = .93; $\alpha3_{post}$ = .95)	Please indicate how much you agree with the following statement.<br>1.Vaccinating against dysomeria is necessary.<br>2.Vaccinating against dysomeria is a good idea.<br>3.Vaccinating against dysomeria is beneficial.<br>4.Vaccinating against dysomeria is safe. (only in Experiment 3)<br>(1 = I strongly disagree, 7 = I strongly agree)	[1,2]
intention to buy genetically modified foods (Exp. 4)	7-point rating scale	If you had the opportunity to buy genetically modified versions of your favourite vegetables, what would you do?(1 = I would definitely not buy them, 7 = I would definitely buy them)	[1,2]
attitude towards genetically modified foods (Exp. 4)	mean score of 7-point rating scales ($\alpha4_{pre}$ = .94; $\alpha4_{post}$ = .95)	Please indicate how much you agree with the following statement.<br>1.Genetically modified foods are necessary.<br>2.Genetically modified foods are a good idea.<br>3.Genetically modified foods are beneficial.<br>4.Genetically modified foods are safe to eat.<br>(1 = I strongly disagree, 7 = I strongly agree)	[1,2]
**moderator variables**			
verbal aggressiveness (Exp. 1; Exp. 2)	mean score of 7-point rating scales (3 items) ($\alpha1$ = .73; $\alpha2$ = .74)	Please indicate how uncharacteristic or characteristic each of the following statements is in describing you.<br>Example item: I tell my friends openly when I disagree with them.	[3]
verbal aggressiveness (Exp. 3; Exp. 4)	mean score of 7-point rating scales (10 items: Factor 1) ($\alpha3$ = .89; $\alpha4$ = .90)	Please indicate how often each statement is true for you personally when you try to influence other persons.<br>Example item: When individuals are very stubborn, I use insults to soften their stubbornness.	[4,5]

need for cognition (Exp. 3; Exp. 4)	mean score of 7-point rating scales (6 items) (α3 = .82; α4 = .86)	Describe the extent to which you agree with each of the following statements.<br>Example item: I would prefer complex to simple problems.<br>(1 = I strongly disagree, 7 = I strongly agree)	6
frequency of social media use (Exp. 1; Exp. 2)	7-point rating scale	How often do you use social media platforms?<br>(1 = never, 7 = very often)	n.a.
issue involvement (Exp. 3; Exp. 4)	mean score of 7-point rating scales (α4 = .66; α4 = .73)	Please indicate how much you agree with the following statements.<br>1. I am interested in the issue of vaccination/genetically modified food.<br>2. I often contemplate about the issue of vaccination/genetically modified food.<br>(1 = I strongly disagree, 7 = I strongly agree)	7
perceived valence (moderator in moderated mediation model in Exp. 1)	7-point rating scale	Now that you have read *name denier* comments and *name advocate* responses in the social media conversation, please rate their contributions.<br>(1 = very negative, 7 = very positive)	8
**mediator variables**			
perceived competence	7-point rating scale	Now that you have read *name denier* comments and *name advocate* responses in the social media conversation, please rate their contributions.<br>(1 = not at all competent, 7 = very competent)	9
perceived expectancy (Exp 1)	mean score of 7-point rating scales (2 items) ($\alpha 2_{\text{advocate argument}}$ = .89; $\alpha 1_{\text{denier argument}}$ = .79; $\alpha 2_{\text{advocate argument}}$ = .95; $\alpha 2_{\text{denier argument}}$ = .87)	Compared to other social media comments, how typical were *name advocate*/*name denier* two comments in the given context?<br>(1 = completely untypical, 7 = completely typical)<br><br>Compared to other social media comments, how expected were *name advocate/name denier* two responses in the given context?<br>(1 = completely unexpected, 7 = completely expected)	10
**additional measures**			
attention (Exp 1; Exp 2)	single item selection	Please think about the social media conversation. What was the conversation about?<br>About the vaccination against dysomeria.<br>About the vaccination against verococci.<br>About the vaccination record of Steve Miller.	1,2

attention 2 (Exp 3; Exp 4)	single item selection	Please select "Almost always true" for this question. (Embedded at the end of the verbal aggressiveness scale)	11
perceived politeness	7-point rating scale	Now that you have read *name denier* comments and *name advocate* responses in the social media conversation, please rate their contributions.<br>(1 = not at all polite, 7 = very polite)	12
perceived authenticity	7-point rating scale	Now that you have read John's two comments and Bill's two responses in the social media conversation, please rate their contributions. I think the conversation was...<br>(1 = not at all realistic, 7 = very realistic)	n.a.
social desirability (Exp 3; Exp 4)	Sum score of responses to three items (α3 = .54/50; α4 = .54/.51)*	Please answer these questions with 'yes' or 'no' as it pertains to you personally.<br>Example item: Would you ever lie to people?<br>(0 = no, 1 = yes)	13
demographics		Please tell us:	
	open question	Your age:	
	single item selection	Your Gender: man; woman; other/non-binary	
	single item selection	Your highest educational qualification: None; High school diploma/GED; Associate’s degree; Bachelor’s degree; Master’s degree; PhD	
additional user comment (Exp. 1)	open question	Imagine you could respond to the social media conversation on #YourVoice yourself. What would you respond? Please enter your response at the bottom of this page.	n.a.

Supplementary Table 5. Overview of measures used in Experiments 1-4.
Reliability of multiple item scales is indicated using Cronbach’s alpha; numbers behind alphas relate to the respective experiments. *alphas are reported for the shortened 3 item version and the four item version (see captions of Supplementary Table 37 and 38 for details).

Experiment	Outcome	Independence of cases	Homogeneity of regression slopes (ANCOVA)	Independence treatment and covariate (ANCOVA)	Homogeneity of variances	Normal distributions
**1**	Attitude	Confirmed by design	Confirmed by ANOVA with interaction of covariate and experimental conditions<br><br>Baseline_Value *Advocate: *p* = .274<br>Baseline_Value *Denier: *p* = .300	Confirmed by ANOVA with covariate as outcome and experimental conditions as factors<br><br>Advocate: *p* = .142 Denier: *p* = .975 Interaction: *p* = .355	Confirmed by variance ratio below 2<br><br>627.487/343.010 = 1.83	Shapiro_Wilk tests were significant for all conditions. We proceeded with F-tests as they are considered robust even for non-normal data[14]
	Intention	Confirmed by design	Baseline_Value *Advocate: *p* = .720<br>Baseline_Value *Denier: *p* = .706	Advocate: *p* = .095 Denier: *p* = .279 Interaction: *p* = .582	823.647/494.660 = 1.67	
**2**	Attitude	Confirmed by design	Baseline_Value *Advocate: *p* = .091<br>Baseline_Value *Denier: ***p* = .049**	Advocate: *p* = .707 Denier: *p* = .248 Interaction: *p* = .614	600.922/384.796 = 1.56	
	Intention	Confirmed by design	Baseline_Value *Advocate: *p* = .378<br>Baseline_Value *Denier: ***p* = .003**	Advocate: *p* = .871 Denier: *p* = .476 Interaction: *p* = .875	681.238/465.265 = 1.46	
**3**	Attitude	Confirmed by design	Baseline_Value *Advocate: *p* = .337<br>Baseline_Value *Denier: *p* = .063	Advocate: *p* = .584 Denier: *p* = .521 Interaction: *p* = .656	667.996/341.301 = 1.96	
	Intention	Confirmed by design	Baseline_Value *Advocate: *p* = .671<br>Baseline_Value *Denier: *p* = .694	Advocate: *p* = .878 Denier: *p* = .775 Interaction: *p* = .388	877.045/526.813 = 1.66	
**4**	Attitude	Confirmed by design	Baseline_Value *Advocate: ***p* = .007**<br>Baseline_Value *Denier: *p* = .194	Advocate: *p* = .284 Denier: *p* = .663 Interaction: *p* = .704	848.088/673.582 = 1.26	
	Intention	Confirmed by design	Baseline_Value *Advocate: *p* = .138<br>Baseline_Value *Denier: *p* = .578	Advocate: *p* = .375 Denier: *p* = .807 Interaction: *p* = .863	1160.664/851.892 = 1.36	

Supplementary Table 6: Assumption tests for GLM including ANCOVA.
Three out of sixteen tests for homogeneity of regression slopes revealed significant results. Using additional scatter plots, we judged differences in slopes to be trivial.

	Experiment 1 (Intention)			Experiment 1 (Attitude)		
	F	*p*	η^2	*F*	*p*	η^2
Denier: Hostile vs. neutral	0.02	.884	<.001	1.16	.281	.002
Advocate: Hostile vs. neutral	5.88	.016	.011	5.31	.022	.004
Denier × Advocate	0.007	.935	<.001	0.90	.342	.002
Control variables:						
Intention / Attitude (baseline)	1114	<.001	.683	804.7	<.001	.609
Degrees of Freedom		516			516	

Supplementary Table 7. ANCOVA results of Experiment 1: Impact of hostility (Advocate and Denier) on intention and attitude controlled for baseline values.

Experiment 1	Model A				Model B			
	Intention				Attitude			
	B	*p*	*SE*	*[95% CI]*	*B*	*p*	*SE*	*[95% CI]*
constant	-6.33	.308	6.28	[-19.53, 5.36]	-4.09	.494	9.02	[-21.13, 14.41]
Advocate: homogeneous vs. heterogeneous	-0.06	.961	1.27	[-2.58, 2.39]	-1.13	.359	1.23	[-3.56, 1.34]
Expectancy	0.03	.668	0.08	[-0.13, 0.21]	0.08	.308	0.10	[-0.12, 0.28]
Advocate: Hostile vs. neutral	5.16	.198	3.53	[-1.45, 12.43]	5.97	.120	4.16	[-2.26, 14.29]
Expectancy × Advocate: Hostile vs. neutral	-0.03	.599	0.05	[-0.12, 0.06]	-0.05	.377	0.05	[-0.16, 0.06]
Intention / Attitude (baseline)	0.93	<.001	0.02	[0.88, 0.98]	0.89	<.001	0.05	[0.79, 0.97]
	Expectancy				Expectancy			
constant	64.53	<.001	3.98	[56.52, 72.38]	53.45	<.001	4.98	[43.15, 63.16]
Advocate: homogeneous vs. heterogeneous	2.02	.346	2.13	[-2.05, 6.14]	2.10	.322	2.11	[-1.91, 6.19]
Intention / Attitude (baseline)	0.04	.406	0.05	[-0.05, 0.13]	0.16	.003	0.06	[0.06, 0.28]
Indirect effects								
Hostile: Impact heterogeneity via expectancy	0.01	-	0.12	[-0.23, 0.30]	0.07	-	0.16	[-0.22, 0.46]
Neutral: Impact heterogeneity via expectancy	-0.05	-	0.09	[-0.27, 0.09]	-0.03	-	0.07	[-0.20, 0.11]
Differences in conditional indirect effects (moderated mediation)	-0.06	-	0.16	[-0.45, 0.20]	-0.10	-	0.19	[-0.56, 0.24]

Supplementary Table 8. Moderated mediator analyses Experiment 1: Impact of hostility (Advocate) on indirect effects of homogeneity of conditions on intention (Model A) and attitude (Model B) via expectancy ratings.
*B*s represent unstandardized coefficients. *CI*s represent 95% confidence intervals of the coefficients. *CI*s and *SE*s (Standard Errors) for direct and indirect effects are bootstrapped (5,000 samples).

Experiment 1	Model 1				Model 2			
	Attitude				Intention			
	B	*p*	*SE*	*[95% CI]*	*B*	*p*	*SE*	*[95% CI]*
Constant	-6.44	.321	7.18	[-20.79, 7.59]	-5.37	.420	7.15	[-19.79, 8.20]
Advocate: Hostile vs. neutral	2.19	.570	3.94	[-5.62, 10.00]	0.14	.972	4.34	[-8.33, 8.90]
Perceived competence advocate	0.13	<.001	0.03	[0.08, 0.20]	0.11	<.001	0.03	[0.06, 0.17]
Intention / Attitude (baseline)	0.84	<.001	0.05	[0.73, 0.93]	0.90	<.001	0.03	[0.84, 0.95]
Denier: Hostile vs. neutral	4.61	.225	4.23	[-3.88, 12.89]	0.48	.905	4.31	[-7.73, 9.13]
Denier × Advocate	-1.76	.461	2.41	[-6.61, 2.97]	0.21	.935	2.54	[-4.92, 5.09]
	Perceived competence				Perceived competence			
Constant	-4.91	.681	13.32	[-30.88, 20.91]	6.01	.605	12.48	[-18.69, 29.84]
Advocate: Hostile vs. neutral	30.78	<.001	6.92	[16.94, 44.22]	30.38	<.001	6.89	[16.80, 43.73]
Intention / Attitude (baseline)	0.41	<.001	0.08	[0.27, 0.57]	0.32	<.001	0.06	[0.21, 0.44]
Denier: Hostile vs. neutral	1.62	.816	7.59	[-13.33, 16.46]	0.20	.977	7.62	[-14.85, 15.33]
Denier × Advocate	-4.25	.333	4.39	[-12.86, 4.52]	-3.83	.383	7.62	[-14.85, 15.33]
Indirect effect								
Impact hostility on intention/attitude via competence	4.15	-	1.32	[1.90, 7.05]	3.30	-	1.06	[1.44, 5.59]
Total effect	6.34	-	3.89	[-1.30, 13.98]	3.44	-	4.08	[-7.88, 8.17]

Supplementary Table 9. Mediator analyses Experiment 1: Impact of hostility (Advocate) on attitude (Model 1) and intention (Model 2) via competence ratings. *B*s represent unstandardized coefficients. *CI*s represent 95% confidence intervals of the coefficients. *CI*s and *SE*s (Standard Errors) for direct and indirect effects are bootstrapped (5,000 samples).

Experiment 1	Model 3				Model 4			
	Attitude				Intention			
	B	*p*	*SE*	*[95% CI]*	*B*	*p*	*SE*	*[95% CI]*
Constant	-3.54	.589	7.24	[-17.75, 10.39]	-2.05	.757	6.99	[-16.33, 11.47]
Denier: Hostile vs. neutral	8.43	.030	4.36	[-0.17, 17.23]	4.33	.288	4.31	[-3.88, 12.99]
Perceived competence advocate	-0.11	<.001	0.02	[-0.16, 0.06]	-0.11	<.001	0.02	[-0.16, -0.06]
Intention / Attitude (baseline)	0.85	<.001	0.05	[0.74, 0.94]	0.90	<.001	0.03	[0.83, 0.94]
Advocate: Hostile vs. neutral	6.87	.071	3.89	[-0.88, 14.60]	4.07	.308	4.13	[-4.01, 12.28]
Advocate × Denier	-2.87	.232	2.43	[-7.68, 1.90]	-0.82	.746	2.50	[-5.74, 4.07]
	Perceived competence				Perceived competence			
Constant	33.17	.019	13.18	[6.99, 59.09]	24.59	.071	13.14	[-1.71, 50.05]
Denier: Hostile vs. neutral	33.52	<.001	8.08	[17.97, 49.52]	35.27	<.001	7.95	[19.96, 50.98]
Intention / Attitude (baseline)	-0.44	<.001	0.08	[-0.59, -0.30]	-0.38	<.001	0.05	[-0.49, -0.28]
Advocate: Hostile vs. neutral	4.95	.547	8.17	[-10.80, 21.60]	5.74	.484	8.14	[-9.87, 22.01]
Advocate × Denier	-4.99	.337	5.18	[-15.40, 5.00]	-5.60	.280	5.13	[-15.71, 4.61]
Indirect effect								
Impact hostility on intention/attitude via competence	-3.60	-	1.16	[-6.02, -1.59]	-3.82	-	1.21	[-6.45, -1.78]
Total effect	4.83	-	3.90	[-2.84, 12.50]	0.50	-	4.10	[-7.54, 8.55]

Supplementary Table 10. Mediator analyses Experiment 1: Impact of hostility (Denier) on attitude (Model 3) and intention (Model 4) via competence ratings.
*B*s represent unstandardized coefficients. *CI*s represent 95% confidence intervals of the coefficients. *CI*s and *SE*s (Standard Errors) for direct and indirect effects are bootstrapped (5,000 samples).

Experiment 1	Model 5				Model 6			
	Intention				Attitude			
	B	*p*	*SE*	*[95% CI]*	*B*	*p*	*SE*	*[95% CI]*
Intercept	1.88	.832	7.76	[-13.62, 16.65]	-10.10	.243	7.55	[-25.12, 4.64]
Advocate: Hostile vs. neutral	2.30	.672	4.88	[-7.08, 11.98]	9.15	.081	4.64	[0.06, 18.16]
Frequency social media use	-0.09	.235	0.08	[-0.25, 0.06]	0.03	.610	0.08	[-0.13, 0.20]
Denier × Frequency social media use	0.01	.818	0.05	[-0.08, 0.11]	-0.04	.408	0.04	[-0.13, 0.05]
Intention / Attitude (baseline)	0.94	<.001	0.02	[0.89, 0.98]	0.89	<.001	0.05	[0.80, 0.98]
Denier: Hostile vs. neutral	0.10	.981	4.26	[-8.14, 8.52]	4.81	.219	4.33	[-4.03, 13.38]
Denier × Advocate	0.01	.996	2.52	[-4.98, 4.85]	-2.31	.348	2.47	[-7.25, 2.67]
Model Parameters								
R^2	.694				.617			
F	$F(6, 514) = 193.9, p < .001$				$F(6, 514) = 137.9, p < .001$			

Supplementary Table 11. Moderator analyses Experiment 1: Impact of hostility (Advocate) on intention (Model 5) and attitude (Model 6) as a function of social media use.
*B*s represent unstandardized coefficients. *CI*s represent 95% confidence intervals of the coefficients. *CI*s and *SE*s (Standard Errors) are bootstrapped (5,000 samples).

Experiment 1	Model 7				Model 8			
	Intention				Attitude			
	B	*p*	*SE*	*[95% CI]*	*B*	*p*	*SE*	*[95% CI]*
Intercept	10.55	.223	8.26	[-5.33, 27.05]	6.24	.462	8.73	[-10.73, 23.82]
Denier: Hostile vs. neutral	-6.59	.220	5.25	[-17.07, 3.59]	-3.27	.525	5.18	[-13.99, 6.47]
Frequency social media use	-0.20	.005	0.08	[-0.35, -0.06]	-0.18	.012	0.07	[-0.30, -0.05]
Denier × Frequency social media use	0.09	.056	0.05	[-0.00, 0.19]	0.11	.018	0.05	[0.02, 0.20]
Intention / Attitude (baseline)	0.94	<.001	0.02	[0.89, 0.98]	0.89	<.001	0.05	[0.80, 0.98]
Advocate: Hostile vs. neutral	3.16	.434	4.11	[-4.71, 11.31]	6.30	.105	3.92	[-1.52, 14.18]
Denier × Advocate	-0.05	.984	2.52	[-5.04, 4.85]	-2.35	.337	2.35	[-7.31, 2.64]
Model Parameters								
R^2	.696				.620			
F	$F(6, 514) = 195.9, p < .001$				$F(6, 514) = 140.0, p < .001$			

Supplementary Table 12. Moderator analyses Experiment 1: Impact of hostility (Denier) on intention (Model 7) and attitude (Model 8) as a function of social media use.
*B*s represent unstandardized coefficients. *CI*s represent 95% confidence intervals of the coefficients. *CI*s and *SE*s (Standard Errors) are bootstrapped (5,000 samples).

Experiment 1	Model 9				Model 10			
	Intention				Attitude			
	B	*p*	*SE*	*[95% CI]*	*B*	*p*	*SE*	*[95% CI]*
Intercept	-7.36	.364	8.39	[-24.52, 8.96]	-11.78	.136	8.16	[-28.03, 4.34]
Advocate: Hostile vs. neutral	5.67	.254	5.01	[-3.78, 15.70]	8.87	.062	4.84	[-0.57, 18.62]
Verbal Aggression	0.05	.555	0.10	[-0.13, 0.25]	0.09	.266	0.10	[-0.10, 0.30]
Advocate × Verbal Aggression	-0.04	.432	0.06	[-0.16, 0.06]	-0.05	.346	0.05	[-0.16, 0.06]
Intention / Attitude (baseline)	0.93	<.001	0.02	[0.88, 0.98]	0.89	<.001	0.05	[0.80, 0.97]
Denier: Hostile vs. neutral	0.39	.925	4.36	[-7.95, 9.01]	4.61	.239	4.35	[-4.17, 13.27]
Denier × Advocate	-0.12	.964	2.57	[-5.20, 4.85]	-2.22	.367	2.48	[-7.12, 2.78]
Model Parameters								
R^2	.688				.617			
F	$F(6, 514) = 189.3, p < .001$				$F(6, 514) = 137.9, p < .001$			

Supplementary Table 13. Moderator analyses Experiment 1: Impact of hostility (Advocate) on intention (Model 9) and attitude (Model 10) as a function of verbal aggression.
*B*s represent unstandardized coefficients. *CI*s represent 95% confidence intervals of the coefficients. *CI*s and *SE*s (Standard Errors) are bootstrapped (5,000 samples).

Experiment 1	Model 11 Intention				Model 12 Attitude			
	B	*p*	*SE*	*[95% CI]*	*B*	*p*	*SE*	*[95% CI]*
Intercept	2.16	.789	8.72	[-14.99, 19.17]	-5.48	.490	8.35	[-22.49, 10.63]
Denier: Hostile vs. neutral	-3.79	.454	5.39	[-14.49, 6.78]	2.99	.536	5.13	[-7.19, 13.05]
Verbal Aggression	-0.13	.123	0.09	[-0.32, 0.03]	-0.03	.703	0.08	[-0.18, 0.13]
Denier × Verbal Aggression	0.08	.146	0.05	[-0.03, 0.19]	0.03	.525	0.05	[-0.07, 0.13]
Intention / Attitude (baseline)	0.94	<.001	0.02	[0.88, 0.98]	0.89	<.001	0.05	[0.80, 0.98]
Advocate: Hostile vs. neutral	3.47	.395	4.19	[-4.51, 11.92]	6.34	.104	3.97	[-1.59, 14.28]
Denier × Advocate	-0.23	.929	2.55	[-5.28, 4.73]	-2.34	.341	2.48	[-7.25, 2.62]
Model Parameters								
R^2	.689				.616			
F	$F(6, 514) = 190.1, p < .001$				$F(6, 514) = 137.64, p < .001$			

Supplementary Table 14. Moderator analyses Experiment 1: Impact of hostility (Denier) on intention (Model 11) and attitude (Model 12) as a function of verbal aggression.

*B*s represent unstandardized coefficients. *CI*s represent 95% confidence intervals of the coefficients. *CI*s and *SE*s (Standard Errors) are bootstrapped (5,000 samples).

	Experiment 2 (Intention)			Experiment 2 (Attitude)		
	F	*p*	η^2	*F*	*p*	η^2
Denier: Hostile vs. neutral	5.13	.024	.017	0.35	.556	.001
Advocate: Hostile vs. none	2.43	.119	.008	6.43	.012	.021
Denier × Advocate	0.01	.907	<.001	0.02	.875	<.001
Control variables:						
Intention / Attitude (baseline)	427.6	<.001	.584	314.1	<.001	.507
Degrees of Freedom		305			305	

Supplementary Table 15. ANCOVA results of Experiment 2: Impact of hostility (Advocate and Denier) on intention and attitude controlled for baseline values.

Experiment 2	Model 1				Model 2			
	Attitude				Intention			
	B	*p*	*SE*	*[95% CI]*	*B*	*p*	*SE*	*[95% CI]*
Constant	7.96	.296	9.51	[-9.87, 26.68]	14.68	.037	7.43	[0.67, 30.12]
Denier: Hostile vs. neutral	4.23	.283	3.75	[-2.85, 11.80]	1.19	.760	3.11	[-4.94, 7.29]
Perceived competence advocate	-0.16	<.001	0.04	[-0.24, -0.09]	-0.17	<.001	0.04	[-0.25, -0.09]
Intention / Attitude (baseline)	0.88	<.001	0.12	[0.64, 1.10]	0.85	<.001	0.07	[0.69, 0.98]
Advocate: Hostile vs. neutral	-2.11	.432	2.52	[-7.03, 2.84]	-1.47	.581	2.46	[-6.25, 3.36]
Advocate × Denier	-0.16	.925	1.68	[-3.54, 3.07]	-0.01	.995	1.66	[-3.27, 3.18]
	Perceived competence				Perceived competence			
Constant	33.38	.013	12.57	[8.17, 58.41]	16.64	.192	12.24	[-8.06, 40.29]
Denier: Hostile vs. neutral	29.14	<.001	6.85	[15.89, 42.78]	28.36	<.001	7.03	[14.59, 42.17]
Intention / Attitude (baseline)	-0.46	<.001	0.08	[-0.62, -0.29]	-0.27	<.001	0.08	[-0.42, -0.10]
Advocate: Hostile vs. neutral	-1.83	.701	4.46	[-10.64, 6.98]	-2.45	.614	4.57	[-11.39, 6.77]
Advocate × Denier	0.71	.813	3.00	[-5.26, 6.55]	1.16	.706	3.05	[-4.95, 7.11]
Indirect effect								
Impact hostility on intention/attitude via competence	-4.72	-	1.57	[-8.15, -2.10]	-4.75	-	1.80	[-8.61, -1.78]
Total effect	-0.49	-	3.97	[-8.29, 7.32]	-3.56	-	3.89	[-11.34, 4.22]

Supplementary Table 16. Mediator analyses Experiment 2: Impact of hostility (Denier) on attitude (Model 1) and intention (Model 2) via competence ratings. *B*s represent unstandardized coefficients. *CI*s represent 95% confidence intervals of the coefficients. *CI*s and *SE*s (Standard Errors) for direct and indirect effects are bootstrapped (5,000 samples).

Experiment 2	Model 3				Model 4			
	Intention				Attitude			
	B	*p*	*SE*	*[95% CI]*	*B*	*p*	*SE*	*[95% CI]*
Intercept	15.10	.139	9.15	[-1.54, 35.08]	3.34	.754	9.62	[-15.35, 22.46]
Denier: Hostile vs. neutral	-4.71	.456	4.46	[-13.39, 3.99]	0.14	.982	6.68	[-12.71, 13.76]
Frequency social media use	-0.05	.615	0.08	[-0.20, 0.11]	-0.02	.814	0.09	[-0.21, 0.17]
Denier × Frequency social media use	0.02	.812	0.05	[-0.09, 0.11]	-0.01	.900	0.06	[-0.13, 0.11]
Intention / Attitude (baseline)	0.91	<.001	0.07	[0.75, 1.03]	0.97	<.001	0.12	[0.73, 1.19]
Advocate: Hostile vs. neutral	-1.02	.715	2.61	[-6.13, 4.03]	-1.81	.517	2.61	[-7.01, 3.29]
Denier × Advocate	-0.22	.899	1.75	[-3.70, 3.24]	-0.26	.880	1.75	[-3.74, 3.22]
Model Parameters								
R^2	.514				.514			
F	$F(6, 303) = 71.51, p < .001$				$F(6, 303) = 53.39, p < .001$			

Supplementary Table 17. Moderator analyses Experiment 2: Impact of hostility (Denier) on intention (Model 3) and attitude (Model 4) as a function of social media use.
*B*s represent unstandardized coefficients. *CI*s represent 95% confidence intervals of the coefficients. *CI*s and *SE*s (Standard Errors) are bootstrapped (5,000 samples).

Experiment 2	Model 5 Intention				Model 6 Attitude			
	B	*p*	*SE*	*[95% CI]*	*B*	*p*	*SE*	*[95% CI]*
Intercept	20.29	.034	11.17	[-0.41, 43.40]	8.14	.415	11.36	[-14.06, 31.11]
Denier: Hostile vs. neutral	-8.97	.110	4.74	[-18.00, 0.37]	-3.10	.587	5.58	[-13.92, 7.98]
Verbal Aggression	-0.16	.178	0.14	[-0.46, 0.12]	-0.12	.322	0.13	[-0.37, 0.14]
Denier × Verbal Aggression	0.11	.166	0.09	[-0.06, 0.28]	0.06	.452	0.09	[-0.11, 0.23]
Intention / Attitude (baseline)	0.89	<.001	0.07	[0.73, 1.02]	0.96	<.001	0.12	[0.72, 1.17]
Advocate: Hostile vs. neutral	-0.83	.767	2.71	[-6.08, 4.46]	-1.37	.627	2.62	[-6.57, 3.73]
Denier × Advocate	-0.24	.893	1.75	[-3.72, 3.17]	-0.50	.777	1.76	[-4.03, 2.90]
Model Parameters								
R^2	.588				.514			
F	$F(6, 303) = 71.95, p < .001$				$F(6, 303) = 53.42, p < .001$			

Supplementary Table 18. Moderator analyses Experiment 2: Impact of hostility (Denier) on intention (Model 5) and attitude (Model 6) as a function of verbal aggression.
*B*s represent unstandardized coefficients. *CI*s represent 95% confidence intervals of the coefficients. *CI*s and *SE*s (Standard Errors) are bootstrapped (5,000 samples).

	Experiment 3 (Intention)			Experiment 3 (Attitude)		
	F	*p*	η^2	*F*	*p*	η^2
Denier: Hostile vs. neutral	6.59	.010	.006	14.10	<.001	.012
Advocate: Hostile vs. neutral vs. none	15.85	.001	.026	18.88	<.001	.031
Denier × Advocate	3.38	.034	.006	2.22	.109	.004
Control variables:						
Intention / Attitude (baseline)	1991.1	< .001	.625	917.4	< .001	.435
Degrees of Freedom		1193			1193	

Supplementary Table 19. ANCOVA results of Experiment 3: Impact of hostility (Advocate and Denier) on intention and attitude controlled for baseline values.

Experiment 3	Model 1				Model 2			
	Attitude				Intention			
	B	*p*	*SE*	*[95% CI]*	*B*	*p*	*SE*	*[95% CI]*
Constant	15.07	<.001	5.56	[4.21, 25.98]	1.38	.650	4.00	[-6.47, 9.39]
Advocate: Hostile vs. neutral	-2.13	.503	3.20	[-8.41, 4.04]	-0.55	.865	3.31	[-7.11, 5.85]
Perceived competence advocate	0.09	<.001	0.02	[0.05, 0.14]	0.08	<.001	0.02	[0.04, 0.12]
Intention / Attitude (baseline)	0.78	<.001	0.06	[0.66, 0.89]	0.90	<.001	0.04	[0.83, 0.97]
Denier: Hostile vs. neutral	-2.82	.047	1.40	[-5.62, 0.16]	-0.70	.631	1.47	[-3.57, 2.19]
Denier × Advocate	1.19	.554	1.98	[-2.74, 5.05]	-0.08	.967	2.03	[-4.05, 3.85]
	Perceived competence				Perceived competence			
Constant	22.82	<.001	6.98	[8.54, 36.45]	25.20	<.001	6.15	[13.27, 37.29]
Advocate: Hostile vs. neutral	22.91	<.001	6.94	[9.28, 36.35]	24.49	<.001	6.97	[10.59, 38.11]
Intention / Attitude (baseline)	0.35	<.001	0.06	[0.23, 0.47]	0.31	<.001	0.04	[0.22, 0.40]
Denier: Hostile vs. neutral	1.09	.716	3.06	[4.82, 6.96]	1.72	.565	3.03	[-4.18, 7.62]
Denier × Advocate	-1.90	.655	4.29	[-10.15, 6.55]	-2.76	.515	4.30	[-11.23, 5.81]
Indirect effect								
Impact hostility on intention/attitude via competence	2.10	-	0.81	[0.73, 3.87]	1.90	-	0.73	[0.68, 3.52]
Total effect	-0.03	-	3.21	[-6.33, 6.28]	1.35	-	3.26	[-5.10, 7.75]

Supplementary Table 20. Mediator analyses Experiment 3: Impact of hostility (Advocate) on attitude (Model 1) and intention (Model 2) via competence ratings. *B*s represent unstandardized coefficients. *CI*s represent 95% confidence intervals of the coefficients. *CI*s and *SE*s (Standard Errors) for direct and indirect effects are bootstrapped (5,000 samples).

Experiment 3	Model 3				Model 4			
	Attitude				Intention			
	B	*p*	*SE*	*[95% CI]*	*B*	*p*	*SE*	*[95% CI]*
Constant	23.33	<.001	5.30	[12.85, 33.30]	7.95	.011	3.79	[0.48, 15.54]
Denier: Hostile vs. neutral	-1.78	.293	2.01	[-5.73, 2.17]	-2.57	.127	1.87	[-6.23, 1.03]
Perceived competence advocate	-0.16	<.001	0.02	[-0.21, -0.12]	-0.12	<.001	0.02	[-0.16, -0.08]
Intention / Attitude (baseline)	0.72	<.001	0.05	[0.62, 0.82]	0.89	<.001	0.03	[0.84, 0.95]
Advocate: Hostile vs. absent	1.40	.699	3.68	[-5.62, 8.67]	-1.62	.654	3.76	[-8.83, 5.68]
Advocate: Hostile vs. absent × Denier	3.20	.163	2.40	[-1.47, 7.71]	4.87	.033	2.41	[0.18, 9.59]
Advocate: Neutral vs. absent	4.24	.246	3.79	[-3.24, 11.63]	1.50	.680	3.57	[-5.62, 8.36]
Advocate: Neutral vs. absent × Denier	2.11	.363	2.43	[-2.66, 6.98]	3.24	.159	2.32	[-1.28, 7.92]
	Perceived competence				Perceived competence			
Constant	22.34	<.001	5.65	[11.69, 33.98]	22.12	<.001	4.85	[12.74, 31.91]
Denier: Hostile vs. neutral	28.82	<.001	2.53	[23.73, 33.74]	28.90	<.001	2.44	[23.88, 33.53]
Intention / Attitude (baseline)	-0.37	<.001	0.05	[-0.47, -0.29]	-0.37	<.001	0.03	[-0.42, -0.30]
Advocate: Hostile vs. absent	9.90	.123	5.81	[-1.30, 21.11]	11.13	.080	5.69	[0.04, 22.00]
Advocate: Hostile vs. absent × Denier	-3.73	.361	3.83	[-11.17, 3.67]	-4.44	.271	3.73	[-11.[54]73, 2.79]
Advocate: Neutral vs. absent	26.70	<.001	6.50	[14.20, 39.11]	26.18	<.001	6.38	[13.62, 38.49]
Advocate: Neutral vs. absent × Denier	-16.07	<.001	4.11	[-24.10, -8.00]	-15.89	<.001	4.05	[-23.76, -7.95]
Indirect effect								
Impact hostility on intention/attitude via competence	-4.74	-	0.71	[-6.13, -3.41]	-3.37	-	0.66	[-4.69, -2.15]
Total effect	-6.52	-	1.69	[-9.83, -3.20]	-5.94	-	1.65	[-9.18, -2.71]

Supplementary Table 21. Mediator analyses Experiment 3: Impact of hostility (Denier) on attitude (Model 3) and intention (Model 4) via competence ratings. *B*s represent unstandardized coefficients. *CI*s represent 95% confidence intervals of the coefficients. *CI*s and *SE*s (Standard Errors) for direct and indirect effects are bootstrapped (5,000 samples).

Experiment 3	Model 5				Model 6			
	Intention				Attitude			
	B	*p*	*SE*	*[95% CI]*	*B*	*p*	*SE*	*[95% CI]*
Intercept	4.92	.130	4.56	[-3.72, 14.38]	18.13	<.001	6.02	[6.48, 30.24]
Advocate: Hostile vs. neutral	-0.90	.802	3.67	[-8.12, 6.29]	-1.23	.729	3.50	[-8.29, 5.59]
Verbal Aggression	-0.06	.150	0.04	[-0.14, 0.02]	-0.03	.466	0.04	[-0.10, 0.04]
Advocate × Verbal Aggression	0.08	.155	0.06	[-0.03, 0.20]	0.04	.434	0.06	[-0.06, 0.15]
Intention / Attitude (baseline)	0.93	<.001	0.03	[0.86, 0.99]	0.81	<.001	0.06	[0.69, 0.92]
Denier: Hostile vs. neutral	-0.74	.614	1.50	[-3.70, 2.21]	-2.81	.053	1.42	[-5.64, -0.11]
Denier × Advocate	-0.09	.965	2.05	[-4.16, 3.92]	1.12	.586	2.02	[-2.93, 5.13]
Model Parameters								
R^2	.679				.533			
F	$F(6, 793) = 279.9, p < .001$				$F(6, 793) = 150.60, p < .001$			

Supplementary Table 22. Moderator analyses Experiment 3: Impact of hostility (Advocate) on intention (Model 5) and attitude (Model 6) as a function of verbal aggression.
*B*s represent unstandardized coefficients. *CI*s represent 95% confidence intervals of the coefficients. *CI*s and *SE*s (Standard Errors) are bootstrapped (5,000 samples).

Experiment 3	Model 7				Model 8			
	Intention				Attitude			
	B	*p*	*SE*	*[95% CI]*	*B*	*p*	*SE*	*[95% CI]*
Intercept	9.01	.015	4.40	[0.52, 17.82]	19.97	<.001	5.85	[8.53, 31.20]
Denier: Hostile vs. neutral	-7.48	<.001	2.18	[-11.85, -3.25]	-6.07	.004	2.46	[-10.96, -1.35]
Verbal Aggression	-0.15	.054	0.10	[-0.36, 0.03]	-0.01	.914	0.10	[-0.20, 0.17]
Denier × Verbal Aggression	0.06	.222	0.06	[-0.05, 0.19]	-0.02	.690	0.06	[-0.14, 0.10]
Intention / Attitude (baseline)	0.94	<.001	0.03	[0.89, 0.99]	0.78	<.001	0.05	[0.68, 0.88]
Advocate: Hostile vs. absent	-2.51	.494	3.87	[-10.07, 4.91]	-0.11	.978	3.88	[-7.51, 7.59]
Advocate: Hostile vs. absent × Denier	5.19	.026	2.50	[0.35, 10.18]	3.75	.116	2.54	[-1.31, 8.57]
Advocate: Neutral vs. absent	-1.48	.688	3.66	[-8.74, 5.74]	-0.14	.970	3.91	[-7.75, 7.61]
Advocate: Neutral vs. absent × Denier	5.07	.030	2.41	[0.29, 9.93]	4.76	.047	2.53	[-0.24, 9.80]
Model Parameters								
R^2	.633				.451			
F	$F(8, 1191) = 256.7, p < .001$				$F(8, 1191) = 122.2, p < .001$			

Supplementary Table 23. Moderator analyses Experiment 3: Impact of hostility (Denier) on intention (Model 7) and attitude (Model 8) as a function of verbal aggression.

*B*s represent unstandardized coefficients. *CI*s represent 95% confidence intervals of the coefficients. *CI*s and *SE*s (Standard Errors) are bootstrapped (5,000 samples).

Experiment 3	Model 9 Intention				Model 10 Attitude			
	B	*p*	*SE*	*[95% CI]*	*B*	*p*	*SE*	*[95% CI]*
Intercept	2.08	.594	5.73	[-8.98, 13.63]	12.56	.002	5.70	[1.28, 23.46]
Advocate: Hostile vs. neutral	2.12	.663	5.60	[-9.01, 13.38]	3.64	.445	5.92	[-7.81, 15.17]
Need for Cognition	0.02	.586	0.04	[-0.06, 0.11]	0.08	.049	0.04	[0.01, 0.15]
Advocate × Need for Cognition	-0.01	.831	0.07	[-0.14, 0.12]	-0.06	.304	0.06	[-0.18, 0.07]
Intention / Attitude (baseline)	0.93	<.001	0.03	[0.86, 0.99]	0.80	<.001	0.06	[0.69, 0.92]
Denier: Hostile vs. neutral	-0.57	.700	1.49	[-3.51, 2.33]	-2.72	.059	1.42	[-5.60, 0.01]
Denier × Advocate	-0.31	.882	2.06	[-4.31, 3.71]	0.98	.630	1.99	[-2.99, 4.90]
Model Parameters								
R^2	.678				.535			
F	$F(6,793) = 278.8.9, p < .001$				$F(6,793) = 151.8, p < .001$			

Supplementary Table 24. Moderator analyses Experiment 3: Impact of hostility (Advocate) on intention (Model 9) and attitude (Model 10) as a function of need for cognition.

*B*s represent unstandardized coefficients. *CI*s represent 95% confidence intervals of the coefficients. *CI*s and *SE*s (Standard Errors) are bootstrapped (5,000 samples).

Experiment 3	Model 11				Model 12			
	Intention				Attitude			
	B	*p*	*SE*	*[95% CI]*	*B*	*p*	*SE*	*[95% CI]*
Intercept	12.09	.041	7.13	[-1.80, 26.06]	22.82	<.001	8.01	[7.16, 38.23]
Denier: Hostile vs. neutral	-11.95	.001	4.46	[-20.89, -3.44]	-11.30	.003	4.20	[-19.53, -3.22]
Need for Cognition	-0.10	.217	0.09	[-0.28, 0.08]	-0.04	.633	0.09	[-0.20, 0.13]
Denier × Need for Cognition	0.09	.073	0.06	[-0.03, 0.21]	0.07	.171	0.06	[-0.04, 0.18]
Intention / Attitude (baseline)	0.93	<.001	0.03	[0.88, 0.98]	0.78	<.001	0.05	[0.68, 0.87]
Advocate: Hostile vs. absent	-3.04	.408	3.85	[-10.49, 4.39]	-0.47	.901	3.86	[-7.74, 7.18]
Advocate: Hostile vs. absent × Denier	5.52	.018	2.48	[0.68, 10.41]	4.02	.092	2.52	[-1.01, 8.76]
Advocate: Neutral vs. absent	-1.52	.680	3.65	[-8.85, 5.72]	-0.21	.955	3.91	[-7.81, 7.48]
Advocate: Neutral vs. absent × Denier	5.07	.030	2.40	[0.34, 9.84]	4.76	.046	2.52	[-0.22, 9.77]
Model Parameters								
R^2	.632				.454			
F	$F(8, 1191) = 256, p < .001$				$F(8, 1191) = 123.6, p < .001$			

Supplementary Table 25. Moderator analyses Experiment 3: Impact of hostility (Denier) on intention (Model 11) and attitude (Model 12) as a function of need for cognition.
*B*s represent unstandardized coefficients. *CI*s represent 95% confidence intervals of the coefficients. *CI*s and *SE*s (Standard Errors) are bootstrapped (5,000 samples).

Experiment 3	Model 13 Intention				Model 14 Attitude			
	B	*p*	*SE*	*[95% CI]*	*B*	*p*	*SE*	*[95% CI]*
Intercept	3.58	.298	4.48	[-5.14, 12.56]	16.43	<.001	5.83	[5.26, 27.97]
Advocate: Hostile vs. neutral	-2.25	.583	4.30	[-10.75, 6.08]	-0.26	.950	4.47	[-9.40, 8.27]
Issue Involvement	-0.00	.964	0.03	[-0.06, 0.06]	0.01	.615	0.03	[-0.04, 0.07]
Advocate × Issue Involvement	0.06	.155	0.04	[-0.02, 0.15]	<0.01	.933	0.05	[-0.08, 0.09]
Intention / Attitude (baseline)	0.92	<.001	0.03	[0.85, 0.99]	0.81	<.001	0.06	[0.69, 0.92]
Denier: Hostile vs. neutral	-0.56	.701	1.49	[-3.50, 2.42]	-2.73	.059	1.43	[-5.63, 0.02]
Denier × Advocate	-0.27	.898	2.04	[-4.27, 3.73]	1.03	.615	2.02	[-3.04, 4.99]
Model Parameters								
R^2	.680				.680			
F	$F(6, 793) = 280.7, p < .001$				$F(6, 793) = 150.6, p < .001$			

Supplementary Table 26. Moderator analyses Experiment 3: Impact of hostility (Advocate) on intention (Model 13) and attitude (Model 14) as a function of issue involvement.

*B*s represent unstandardized coefficients. *CI*s represent 95% confidence intervals of the coefficients. *CI*s and *SE*s (Standard Errors) are bootstrapped (5,000 samples).

Experiment 3	Model 15 Intention				Model 16 Attitude			
	B	*p*	*SE*	*[95% CI]*	*B*	*p*	*SE*	*[95% CI]*
Intercept	0.47	.920	5.32	[-10.21, 10.56]	12.06	.016	6.68	[-1.69, 24.57]
Denier: Hostile vs. neutral	-2.96	.296	3.26	[-9.33, 3.32]	-1.91	.510	3.40	[-8.55, 5.05]
Issue Involvement	0.08	.162	0.06	[-0.03, 0.21]	0.13	.034	0.07	[0.01, 0.27]
Denier × Issue Involvement	-0.05	.197	0.04	[-0.13, 0.03]	-0.08	.051	0.04	[-0.16, 0.00]
Intention / Attitude (baseline)	0.93	<.001	0.03	[0.88, 0.99]	0.78	<.001	0.05	[0.68, 0.88]
Advocate: Hostile vs. absent	-2.85	.439	3.85	[-10.35, 4.62]	-0.13	.972	3.86	[-7.43, 7.44]
Advocate: Hostile vs. absent × Denier	5.35	.022	2.49	[0.46, 10.24]	3.76	.115	2.54	[-1.26, 8.60]
Advocate: Neutral vs. absent	-1.54	.676	3.64	[-8.78, 5.57]	-0.10	.978	3.87	[-7.60, 7.46]
Advocate: Neutral vs. absent × Denier	5.08	.030	2.40	[0.37, 9.84]	4.71	.049	2.51	[-0.20, 9.65]
Model Parameters								
R^2	.631				.452			
F	$F(8, 1191) = 254.9, p < .001$				$F(8, 1191) = 122.7, p < .001$			

Supplementary Table 27. Moderator analyses Experiment 3: Impact of hostility (Denier) on intention (Model 15) and attitude (Model 16) as a function of issue involvement.

*B*s represent unstandardized coefficients. *CI*s represent 95% confidence intervals of the coefficients. *CI*s and *SE*s (Standard Errors) are bootstrapped (5,000 samples).

	Experiment 4 (Intention)			Experiment 4 (Attitude)		
	F	*p*	η^2	*F*	*p*	η^2
Denier: Hostile vs. neutral	4.86	.028	.004	0.38	.538	<.001
Advocate: Hostile vs. neutral vs. none	31.05	<.001	.050	47.06	<.001	.073
Denier × Advocate	1.61	.200	.003	0.79	.453	.001
Control variables:						
Intention / Attitude (baseline)	2981.6	<.001	.715	2788.4	<.001	.701
Degrees of Freedom		1188			1188	

Supplementary Table 28: ANCOVA results of Experiment 4: Impact of hostility (Advocate and Denier) on intention and attitude controlled for baseline values.

Experiment 4	Model 1				Model 2			
	Attitude				Intention			
	B	*p*	*SE*	*[95% CI]*	*B*	*p*	*SE*	*[95% CI]*
Constant	5.64	.046	2.65	[0.52, 10.89]	8.86	.002	2.59	[3.83, 13.96]
Advocate: Hostile vs. neutral	-4.25	.214	3.60	[-11.39, 2.75]	-7.57	.035	3.73	[-14.81, -0.23]
Perceived competence advocate	0.13	<.001	0.02	[0.09, 0.17]	0.15	<.001	0.02	[0.11, 0.19]
Intention / Attitude (baseline)	0.83	<.001	0.02	[0.78, 0.87]	0.82	<.001	0.02	[0.78, 0.86]
Denier: Hostile vs. neutral	-0.74	.625	1.38	[-3.43, 1.96]	-2.21	.164	1.42	[-4.95, 0.62]
Denier × Advocate	2.10	.324	2.16	[-2.12, 6.40]	2.74	.223	2.23	[-1.73, 7.03]
	Perceived competence				Perceived competence			
Constant	41.63	<.001	5.36	[31.06, 52.42]	48.02	<.001	4.97	[38.27, 57.88]
Advocate: Hostile vs. neutral	25.47	<.001	6.54	[12.64, 38.12]	24.54	<.001	6.61	[11.52, 37.34]
Intention / Attitude (baseline)	0.19	<.001	0.04	[0.11, 0.27]	0.11	<.001	0.03	[0.04, 0.17]
Denier: Hostile vs. neutral	-1.65	.555	2.99	[-7.62, 4.21]	-1.77	.530	3.00	[-7.68, 4.08]
Denier × Advocate	-1.58	.689	4.00	[-9.41, 6.24]	-1.22	.759	4.04	[-9.04, 6.64]
Indirect effect								
Impact hostility on intention/attitude via competence	3.21	-	1.03	[1.42, 5.47]	3.62	-	1.13	[1.59, 5.93]
Total effect	-1.04	-	3.47	[-7.85, 5.77]	-3.94	-	3.67	[-11.15, 3.26]

Supplementary Table 29. Mediator analyses Experiment 4: Impact of hostility (Advocate) on attitude (Model 1) and intention (Model 2) via competence ratings. *B*s represent unstandardized coefficients. *CI*s represent 95% confidence intervals of the coefficients. *CI*s and *SE*s (Standard Errors) for direct and indirect effects are bootstrapped (5,000 samples).

Experiment 4	Model 3				Model 4			
	Attitude				Intention			
	B	*p*	*SE*	*[95% CI]*	*B*	*p*	*SE*	*[95% CI]*
Constant	2.35	.361	2.42	[-2.42, 7.11]	9.14	<.001	2.76	[3.57, 14.43]
Denier: Hostile vs. neutral	1.76	.279	1.61	[-1.45, 4.96]	0.13	.942	1.99	[-3.70, 4.13]
Perceived competence advocate	-0.09	<.001	0.02	[-0.13, -0.05]	-0.11	<.001	0.02	[-0.15, -0.07]
Intention / Attitude (baseline)	0.86	<.001	0.02	[0.83, 0.90]	0.83	<.001	0.02	[0.80, 0.87]
Advocate: Hostile vs. absent	7.43	.028	3.15	[1.15, 13.74]	6.44	.084	3.53	[-0.26, 13.45]
Advocate: Hostile vs. absent × Denier	0.25	.906	2.02	[-3.69, 4.24]	1.02	.665	2.31	[-3.54, 5.54]
Advocate: Neutral vs. absent	7.54	.026	3.47	[0.68, 14.46]	3.86	.301	3.97	[-3.76, 11.68]
Advocate: Neutral vs. absent × Denier	1.42	.505	2.17	[-2.76, 5.77]	2.70	.254	2.48	[-2.25, 7.49]
	Perceived competence				Perceived competence			
Constant	3.90	.406	4.63	[-4.89, 12.89]	-1.01	.824	4.46	[-9.57, 7.96]
Denier: Hostile vs. neutral	38.17	<.001	2.56	[33.08, 43.03]	38.33	<.001	2.61	[33.14, 43.37]
Intention / Attitude (baseline)	-0.24	<.001	0.03	[-0.31, -0.18]	-0.19	<.001	0.03	[-0.24, -0.14]
Advocate: Hostile vs. absent	7.27	.237	6.06	[-4.76, 19.06]	6.87	.266	6.07	[-5.27, 18.49]
Advocate: Hostile vs. absent × Denier	-5.95	.126	3.83	[-13.36, 1.53]	-6.04	.122	3.80	[-13.36, 1.45]
Advocate: Neutral vs. absent	18.38	.003	6.43	[5.75, 30.67]	19.18	.002	6.49	[6.43, 31.79]
Advocate: Neutral vs. absent × Denier	-13.48	.001	3.89	[-20.99, -5.79]	-14.05	<.001	3.93	[-21.69, -6.22]
Indirect effect								
Impact hostility on intention/attitude via competence	-3.44	-	0.74	[-5.00, -2.04]	-4.27	-	0.80	[-5.90, -2.72]
Total effect	-1.69	-	1.52	[-4.67, 1.30]	-4.14	-	1.69	[-7.45, -0.83]

Supplementary Table 30. Mediator analyses Experiment 4: Impact of hostility (Denier) on attitude (Model 3) and intention (Model 4) via competence ratings. *B*s represent unstandardized coefficients. *CI*s represent 95% confidence intervals of the coefficients. *CI*s and *SE*s (Standard Errors) for direct and indirect effects are bootstrapped (5,000 samples).

Experiment 4	Model 5 Intention				Model 6 Attitude			
	B	*p*	*SE*	*[95% CI]*	*B*	*p*	*SE*	*[95% CI]*
Intercept	16.28	<.001	2.77	[10.73, 21.70]	10.93	<.001	2.79	[5.46, 16.35]
Advocate: Hostile vs. neutral	-3.82	.329	4.14	[-11.86, 4.42]	0.11	.977	3.67	[-7.14, 7.20]
Verbal Aggression	-0.02	.622	0.04	[-0.10, 0.06]	-0.00	.758	0.04	[-0.09, 0.07]
Advocate × Verbal Aggression	>-0.01	.942	0.06	[-0.14, 0.11]	-0.05	.389	0.07	[-0.18, 0.07]
Intention / Attitude (baseline)	0.84	<.001	0.02	[0.80, 0.88]	0.85	<.001	0.02	[0.81, 0.90]
Denier: Hostile vs. neutral	-2.44	.140	1.48	[-5.26, 0.54]	-0.92	.553	1.44	[-3.73, 1.88]
Denier × Advocate	2.51	.279	2.30	[-2.20, 6.97]	1.86	.396	2.22	[-2.52, 6.23]
Model Parameters								
R^2	.720				.681			
F	$F(6, 788) = 337.4, p < .001$				$F(6, 788) = 280.7, p < .001$			

Supplementary Table 31. Moderator analyses Experiment 4: Impact of hostility (Advocate) on intention (Model 5) and attitude (Model 6) as a function of verbal aggression.
*B*s represent unstandardized coefficients. *CI*s represent 95% confidence intervals of the coefficients. *CI*s and *SE*s (Standard Errors) are bootstrapped (5,000 samples).

Experiment 4	Model 7 Intention				Model 8 Attitude			
	B	*p*	*SE*	*[95% CI]*	*B*	*p*	*SE*	*[95% CI]*
Intercept	9.49	.004	3.03	[3.28, 15.49]	3.27	.283	2.79	[-2.24, 8.76]
Denier: Hostile vs. neutral	-4.09	.048	1.96	[-7.90, -0.22]	-1.97	.289	1.76	[-5.49, 1.54]
Verbal Aggression	-0.02	.832	0.09	[-0.19, 0.15]	-0.08	.294	0.09	[-0.25, 0.09]
Denier × Verbal Aggression	>-0.01	.992	0.06	[-0.11, 0.11]	0.02	.710	0.05	[-0.09, 0.12]
Intention / Attitude (baseline)	0.86	<.001	0.016	[0.83, 0.89]	0.89	<.001	0.02	[0.85, 0.92]
Advocate: Hostile vs. absent	5.71	.133	3.56	[-1.13, 12.79]	6.89	.044	3.14	[0.72, 13.19]
Advocate: Hostile vs. absent × Denier	1.69	.481	2.34	[-2.98, 6.29]	0.75	.728	2.04	[-3.37, 4.92]
Advocate: Neutral vs. absent	1.78	.638	4.04	[-5.96, 9.64]	6.05	.076	3.55	[-0.95, 13.12]
Advocate: Neutral vs. absent × Denier	4.22	.078	2.51	[-0.74, 9.07]	2.52	.241	2.21	[-1.78, 6.93]
Model Parameters								
R^2	.718				.708			
F	$F(8, 1186) = 377.1, p < .001$				$F(8, 1186) = 359.5, p < .001$			

Supplementary Table 32. Moderator analyses Experiment 4: Impact of hostility (Denier) on intention (Model 7) and attitude (Model 8) as a function of verbal aggression.
*B*s represent unstandardized coefficients. *CI*s represent 95% confidence intervals of the coefficients. *CI*s and *SE*s (Standard Errors) are bootstrapped (5,000 samples).

Experiment 4	Model 9				Model 10			
	Intention				Attitude			
	B	*p*	*SE*	*[95% CI]*	*B*	*p*	*SE*	*[95% CI]*
Intercept	15.73	<.001	3.80	[8.28, 23.26]	9.63	.008	3.47	[2.74, 16.34]
Advocate: Hostile vs. neutral	-6.67	.192	5.38	[-17.11, 3.93]	4.59	.341	4.97	[-5.21, 14.49]
Need for Cognition	<0.01	.912	0.04	[-0.08, 0.09]	0.02	.658	0.03	[-0.05, 0.09]
Advocate × Need for Cognition	0.04	.451	0.06	[-0.08, 0.16]	-0.09	.097	0.06	[-0.21, 0.03]
Intention / Attitude (baseline)	0.83	<.001	0.02	[0.79, 0.87]	0.85	<.001	0.02	[0.81, 0.90]
Denier: Hostile vs. neutral	-2.48	.131	1.48	[-5.34, 0.54]	-0.96	.534	14.29	[-3.72, 18.38]
Denier × Advocate	2.61	.262	2.29	[-2.08, 7.03]	1.85	.397	2.20	[-2.45, 6.20]
Model Parameters								
R^2	.720				.682			
F	$F(6, 788) = 337.9, p < .001$				$F(6, 788) = 281.5, p < .001$			

Supplementary Table 33. Moderator analyses Experiment 4: Impact of hostility (Advocate) on intention (Model 9) and attitude (Model 10) as a function of need for cognition.
*B*s represent unstandardized coefficients. *CI*s represent 95% confidence intervals of the coefficients. *CI*s and *SE*s (Standard Errors) are bootstrapped (5,000 samples).

Experiment 4	Model 11 Intention				Model 12 Attitude			
	B	*p*	*SE*	*[95% CI]*	*B*	*p*	*SE*	*[95% CI]*
Intercept	9.40	.089	5.51	[-1.37, 20.40]	-0.78	.877	5.17	[-10.61, 9.61]
Denier: Hostile vs. neutral	-4.89	.160	3.40	[-11.77, 1.70]	0.29	.927	3.41	[-6.50, 6.74]
Need for Cognition	>-0.01	.979	0.08	[-0.15, 0.15]	0.04	.520	0.07	[-0.10, 0.18]
Denier × Need for Cognition	0.01	.806	0.05	[-0.08, 0.10]	-0.03	.471	0.04	[-0.12, 0.06]
Intention / Attitude (baseline)	0.85	<.001	0.02	[0.82, 0.89]	0.88	<.001	0.01	[0.85, 0.92]
Advocate: Hostile vs. absent	5.71	.133	3.55	[-1.12, 12.78]	6.80	.047	3.15	[0.61, 13.03]
Advocate: Hostile vs. absent × Denier	1.68	.485	2.33	[-2.91, 6.27]	0.78	.719	2.048	[-3.27, 4.87]
Advocate: Neutral vs. absent	1.71	.652	4.04	[-6.17, 9.68]	5.97	.081	3.57	[-1.03, 13.07]
Advocate: Neutral vs. absent × Denier	4.29	.073	2.51	[-0.64, 9.15]	2.58	.232	2.21	[-1.69, 6.98]
Model Parameters								
R^2	.718				.707			
F	$F(8, 1186) = 377.1, p < .001$				$F(8, 1186) = 357.6, p < .001$			

Supplementary Table 34. Moderator analyses Experiment 4: Impact of hostility (Denier) on intention (Model 11) and attitude (Model 12) as a function of need for cognition.

*B*s represent unstandardized coefficients. *CI*s represent 95% confidence intervals of the coefficients. *CI*s and *SE*s (Standard Errors) are bootstrapped (5,000 samples).

Experiment 4	Model 13				Model 14			
	Intention				Attitude			
	B	*p*	*SE*	*[95% CI]*	*B*	*p*	*SE*	*[95% CI]*
Intercept	14.93	<.001	3.09	[8.91, 21.08]	9.50	.003	2.93	[3.78, 15.31]
Advocate: Hostile vs. neutral	-1.99	.642	4.58	[-10.84, 7.01]	1.50	.712	3.98	[-6.49, 9.08]
Issue Involvement	0.02	.546	0.03	[-0.04, 0.09]	0.03	.392	0.03	[-0.03, 0.08]
Advocate × Issue Involvement	-0.04	.376	0.05	[-0.14, 0.06]	-0.05	.225	0.05	[-0.15, 0.04]
Intention / Attitude (baseline)	0.84	<.001	0.02	[0.80, 0.88]	0.85	<.001	0.02	[0.81, 0.89]
Denier: Hostile vs. neutral	-2.49	.131	1.48	[-5.35, 0.51]	-0.96	.535	1.44	[-3.72, 1.84]
Denier × Advocate	2.54	.275	2.30	[-2.16, 7.00]	1.87	.392	2.21	[-2.44, 6.28]
Model Parameters								
R^2	.720				.681			
F	$F(6, 788) = 337.5, p < .001$				$F(6, 788) = 280.3, p < .001$			

Supplementary Table 35. Moderator analyses Experiment 4: Impact of hostility (Advocate) on intention (Model 13) and attitude (Model 14) as a function of issue involvement.
*B*s represent unstandardized coefficients. *CI*s represent 95% confidence intervals of the coefficients. *CI*s and *SE*s (Standard Errors) are bootstrapped (5,000 samples).

Experiment 4	Model 15				Model 16			
	Intention				Attitude			
	B	*p*	*SE*	*[95% CI]*	*B*	*p*	*SE*	*[95% CI]*
Intercept	9.77	.012	3.74	[2.42, 17.08]	5.40	.127	3.40	[-1.15, 12.31]
Denier: Hostile vs. neutral	-4.20	.080	2.39	[-8.87, 0.55]	-3.86	.074	2.18	[-8.21, 0.40]
Issue Involvement	-0.01	.851	0.07	[-0.15, 0.12]	-0.08	.154	0.06	[-0.20, 0.03]
Denier × Issue Involvement	<0.01	.997	0.04	[-0.08, 0.09]	0.05	.159	0.04	[-0.02, 0.13]
Intention / Attitude (baseline)	0.86	<.001	0.02	[0.82, 0.89]	0.88	<.001	0.02	[0.85, 0.92]
Advocate: Hostile vs. absent	5.65	.138	3.57	[-1.24, 12.77]	7.10	.038	3.16	[0.90, 13.44]
Advocate: Hostile vs. absent × Denier	1.75	.467	2.35	[-2.95, 6.39]	0.54	.803	2.05	[-3.52, 4.61]
Advocate: Neutral vs. absent	1.69	.655	4.03	[-6.17, 9.55]	5.95	.081	3.56	[-1.08, 13.13]
Advocate: Neutral vs. absent × Denier	4.30	.073	2.52	[-0.72, 9.16]	2.58	.231	2.22	[-1.69, 6.98]
Model Parameters								
R^2	.718				.707			
F	$F(8, 1186) = 377, p < .001$				$F(8, 1186) = 358.2, p < .001$			

Supplementary Table 36. Moderator analyses Experiment 4: Impact of hostility (Denier) on intention (Model 15) and attitude (Model 16) as a function of issue involvement.
*B*s represent unstandardized coefficients. *CI*s represent 95% confidence intervals of the coefficients. *CI*s and *SE*s (Standard Errors) are bootstrapped (5,000 samples).

Perceived authenticity	Experiment 1			Experiment 2		
	F	*p*	η^2	*F*	*p*	η^2
Denier: Hostile vs. neutral	0.78	.378	<.001	3.58	.059	.021
Advocate: Hostile vs. neutral (none)	2.68	.103	.004	0.28	.594	.001
Denier × Advocate	0.65	.419	.001	0.03	.873	<.001
Degrees of Freedom		517			306	

Supplementary Table 37. Impact of hostility (Denier and Advocate) on perceived authenticity.

	Experiment 3 (Intention)			Experiment 3 (Attitude)			Experiment 3 (Competence Advocate)*			Experiment 3 (Competence Denier)*		
	F	*p*	η^2	*F*	*p*	η^2	*F*	*p*	η^2	*F*	*p*	η^2
Denier: Hostile vs. neutral	10.45	.001	.003	18.91	.<.001	.010	0.71	.401	.001	133.08	<.001	.124
Advocate: Hostile vs. neutral vs. none	7.92	<.001	.005	9.40	<.001	.010	60.27	<.001	.088	4.01	.018	.007
Denier × Advocate	1.44	.237	<.001	1.23	.293	.001	0.90	.342	.002	5.34	.005	.010
Control variables:												
Intention / Attitude (baseline)	2075.21	<.001	.686	931.37	<.001	.492	--	--	--	--	--	--
Degrees of Freedom	921			921			623			623		
	p			*p*			*p*			*p*		
Contrast: Hostile vs. neutral	.509			.396			--			--		
Contrast: Neutral vs. none	<.001			<.001			--			--		
Contrast: Hostile vs. none	.002			.001			--			--		

Supplementary Table 38. Robustness check of Experiment 3: Impact of hostility (Advocate and Denier) on intention and attitude and competence without individuals that respond in highly social desirable manner.
Social desirability was measured with a 3 item version of the BSDS scale[8]. The fourth item of the original scale (Would you smile at people everytime you meet them?) was dropped because of poor fit (corrected item-total correlation: $r = .167$). The overall cronbach‘s alpha of the three item scale was .536. Scores range from 0 to 3. Individuals with a score of 3 (high social desirable response pattern) were excluded in the presented Ancovas. *These Ancovas did not inlcude the Advocate absent condition.

	Experiment 4 (Intention)			Experiment 4 (Attitude)			Experiment 4 (Competence Advocate)*			Experiment 4 (Competence Denier)*		
	F	*p*	η^2	*F*	*p*	η^2	*F*	*p*	η^2	*F*	*p*	η^2
Denier: Hostile vs. neutral	7.66	.006	.002	2.62	.043	.001	1.87	.172	.003	328.05	<.001	.259
Advocate: Hostile vs. neutral vs. none	26.13	<.001	.001	48.77	<.001	.025	94.26	<.001	.132	0.42	.657	<.001
Denier × Advocate	0.75	.474	<.001	0.17	.843	<.001	1.08	.299	.002	4.18	.016	.007
Control variables:												
Intention / Attitude (baseline)	2472.33	<.001	.749	2938.83	<.001	.740	--	--	--	--	--	--
Degrees of Freedom	930			930			618			618		
	p			*p*			*p*			*p*		
Contrast: Hostile vs. neutral	.539			.051			--			--		
Contrast: Neutral vs. none	<.001			<.001			--			--		
Contrast: Hostile vs. none	<.001			<.001			--			--		

Supplementary Table 39. Robustness check of Experiment 4: Impact of hostility (Advocate and Denier) on intention and attitude and competence without individuals that respond in highly social desirable manner.
Social desirability was measured with a 3 item version of the BSDS scale[8]. The fourth item of the original scale (Would you smile at people everytime you meet them?) was dropped because of poor fit (corrected item-total correlation: $r = .173$). The overall cronbach‘s alpha of the three item scale was .536. Scores range from 0 to 3. Individuals with a score of 3 (high social desirable response pattern) were excluded in the presented Ancovas. *These Ancovas did not inlcude the Advocate absent condition.

	Experiment 1 (Intention)			Experiment 1 (Attitude)			Experiment 1 (Competence Advocate)			Experiment 1 (Competence Denier)		
	F	*p*	η^2	*F*	*p*	η^2	*F*	*p*	η^2	*F*	*p*	η^2
Denier: Hostile vs. neutral	<0.01	.969	<.001	1.29	.257	.001	4.91	.027	.008	93.25	<.001	.156
Advocate: Hostile vs. neutral	6.23	.013	.004	5.83	.016	.004	122.23	<.001	.194	1.58	.210	.003
Denier × Advocate	0.05	.830	<.001	0.99	.319	<.001	0.957	.328	.002	1.27	.260	.002
Control variables:												
Intention / Attitude (baseline)	1076.08	<.001	.674	760.87	<.001	.594	--	--	--	--	--	--
Degrees of Freedom	499			499			500			500		

Supplementary Table 40. Robustness check of Experiment 1: Impact of hostility (Advocate and Denier) on intention and attitude and competence without speeders.

	Experiment 2 (Intention)			Experiment 2 (Attitude)			Experiment 2 (Competence Denier)		
	F	*p*	η^2	*F*	*p*	η^2	*F*	*p*	η^2
Denier: Hostile vs. neutral	2.60	.108	.004	0.67	.413	.001	66.57	<.001	.203
Advocate: Hostile vs. none	5.09	.025	.007	5.55	.019	.009	0.06	.806	<.001
Denier × Advocate	0.13	.717	<.001	0.02	.904	<.001	0.21	.651	<.001
Control variables:									
Intention / Attitude (baseline)	421.73	<.001	.616	355.93	<.001	.572	--	--	--
Degrees of Freedom	260			260			261		

Supplementary Table 41. Robustness check of Experiment 2: Impact of hostility (Advocate and Denier) on intention and attitude and competence without speeders.

	Experiment 3 (Intention)			Experiment 3 (Attitude)			Experiment 3 (Competence Advocate)*			Experiment 3 (Competence Denier)*		
	F	*p*	η^2	*F*	*p*	η^2	*F*	*p*	η^2	*F*	*p*	η^2
Denier: Hostile vs. neutral	7.17	.008	.002	13.99	<.001	.007	0.05	.822	<.001	172.30	<.001	.129
Advocate: Hostile vs. neutral vs. none	16.94	<.001	.011	20.19	<.001	.020	84.43	<.001	.099	1.08	.339	.002
Denier × Advocate	3.55	.029	.002	2.07	.127	.002	0.36	.551	<.001	6.66	.001	.010
Control variables:												
Intention / Attitude (baseline)	1837	<.001	.607	804.11	<.001	.401	--	--	--	--	--	--
Degrees of Freedom	1140			1140			772			1141		
	p			*p*			*p*			*p*		
Contrast: Hostile vs. neutral	.831			.517			--			--		
Contrast: Neutral vs. none	<.001			<.001			--			--		
Contrast: Hostile vs. none	<.001			<.001			--			--		

Supplementary Table 42. Robustness check of Experiment 3: Impact of hostility (Advocate and Denier) on intention and attitude and competence without speeders.

	Experiment 4 (Intention)			Experiment 4 (Attitude)			Experiment 4 (Competence Advocate)*			Experiment 4 (Competence Denier)*		
	F	*p*	η^2	*F*	*p*	η^2	*F*	*p*	η^2	*F*	*p*	η^2
Denier: Hostile vs. neutral	3.55	.060	<.001	0.23	.632	<.001	1.64	.201	.002	381.73	<.001	.246
Advocate: Hostile vs. neutral vs. none	28.35	<.001	.014	43.89	<.001	.023	126.09	<.001	.140	0.34	.712	<.001
Denier × Advocate	1.59	.204	<.001	0.66	.517	<.001	0.04	.833	<.001	5.32	.005	.007
Control variables:												
Intention / Attitude (baseline)	2961.59	<.001	.714	2714.14	<.001	.690	--	--	--	--	--	--
Degrees of Freedom	1156			1156			771			1157		
	p			*p*			*p*			*p*		
Contrast: Hostile vs. neutral	.987			.258			--			--		
Contrast: Neutral vs. none	<.001			<.001			--			--		
Contrast: Hostile vs. none	<.001			<.001			--			--		

Supplementary Table 43. Robustness check of Experiment 4: Impact of hostility (Advocate and Denier) on intention and attitude and competence without speeders.

	Experiment 1 (Intention)			Experiment 1 (Attitude)			Experiment 1 (Competence Advocate)			Experiment 1 (Competence Denier)		
	F	*p*	η^2	*F*	*p*	η^2	*F*	*p*	η^2	*F*	*p*	η^2
Denier: Hostile vs. neutral	0.02	.899	<.001	1.67	.197	.001	7.55	.006	.012	94.91	<.001	.159
Advocate: Hostile vs. neutral	6.24	.013	.004	6.51	.011	.005	126.71	<.001	.200	2.09	.149	.004
Denier × Advocate	0.02	.882	<.001	0.79	.374	<.001	0.571	.450	<.001	0.80	.373	.001
Control variables:												
Intention / Attitude (baseline)	1060.47	<.001	.678	784.97	<.001	.608	--	--	--	--	--	--
Degrees of Freedom	497			497			498			498		

Supplementary Table 44. Robustness check of Experiment 1: Impact of hostility (Advocate and Denier) on intention and attitude and competence without inattentive individuals.

	Experiment 2 (Intention)			Experiment 2 (Attitude)			Experiment 2 (Competence Denier)		
	F	*p*	η^2	*F*	*p*	η^2	*F*	*p*	η^2
Denier: Hostile vs. neutral	5.22	.023	.007	0.30	.582	<.001	97.28	<.001	.242
Advocate: Hostile vs. none	2.31	.130	.003	6.53	.011	.011	0.55	.458	.001
Denier × Advocate	0.02	.882	<.001	0.02	.904	<.001	0.23	.634	<.001
Control variables:									
Intention / Attitude (baseline)	425.65	<.001	.578	311.70	<.001	.501	--	--	--
Degrees of Freedom	303			303			304		

Supplementary Table 45. Robustness check of Experiment 2: Impact of hostility (Advocate and Denier) on intention and attitude and competence without inattentive individuals.

	Experiment 3 (Intention)			Experiment 3 (Attitude)			Experiment 3 (Competence Advocate)*			Experiment 3 (Competence Denier)*		
	F	*p*	η^2	*F*	*p*	η^2	*F*	*p*	η^2	*F*	*p*	η^2
Denier: Hostile vs. neutral	7.26	.007	.002	13.83	<.001	.007	0.01	.946	<.001	167.39	<.001	.123
Advocate: Hostile vs. neutral vs. none	16.42	<.001	.010	17.88	<.001	.017	84.51	<.001	.097	2.60	.075	.004
Denier × Advocate	3.55	.029	.002	2.21	.110	.002	0.44	.506	<.001	7.00	<.001	.010
Control variables:												
Intention / Attitude (baseline)	1909.54	<.001	.610	873.19	<.001	.416	--	--	--	--	--	--
Degrees of Freedom	1174			1174			784			1175		
	p			*p*			*p*			*p*		
Contrast: Hostile vs. neutral	.388			.243			--			--		
Contrast: Neutral vs. none	<.001			<.001			--			--		
Contrast: Hostile vs. none	<.001			<.001			--			--		

Supplementary Table 46. Robustness check of Experiment 3: Impact of hostility (Advocate and Denier) on intention and attitude and competence without inattentive individuals.

	Experiment 4 (Intention)			Experiment 4 (Attitude)			Experiment 4 (Competence Advocate)*			Experiment 4 (Competence Denier)*		
	F	*p*	η^2	*F*	*p*	η^2	*F*	*p*	η^2	*F*	*p*	η^2
Denier: Hostile vs. neutral	4.85	.028	.001	0.19	.662	<.001	1.20	.274	<.001	377.94	<.001	.243
Advocate: Hostile vs. neutral vs. none	34.91	<.001	.016	48.06	<.001	.024	126.99	<.001	.140	0.203	.817	<.001
Denier × Advocate	1.56	.211	<.001	0.76	.469	<.001	0.29	.589	<.001	5.51	.004	.007
Control variables:												
Intention / Attitude (baseline)	3013.52	<.001	.708	2709.90	<.001	.682	--	--	--	--	--	--
Degrees of Freedom	1168			1168			776			1168		
	p			*p*			*p*			*p*		
Contrast: Hostile vs. neutral	.973			.080			--			--		
Contrast: Neutral vs. none	<.001			<.001			--			--		
Contrast: Hostile vs. none	<.001			<.001			--			--		

Supplementary Table 47. Robustness check of Experiment 4: Impact of hostility (Advocate and Denier) on intention and attitude and competence without inattentive individuals.

Supplementary References

1. Schmid, P. & Betsch, C. Effective strategies for rebutting science denialism in public discussions. *Nat Hum Behav* **3**, 931–939 (2019).
2. Schmid, P., Schwarzer, M. & Betsch, C. Weight-of-Evidence Strategies to Mitigate the Influence of Messages of Science Denialism in Public Discussions. *J Cogn* **3**, 36 (2020).
3. Webster, G. D. *et al.* The Brief Aggression Questionnaire: Structure, Validity, Reliability, and Generalizability. *Journal of Personality Assessment* **97**, 638–649 (2015).
4. Infante, D. A. & Wigley, C. J. Verbal aggressiveness: An interpersonal model and measure. *Communication Monographs* **53**, 61–69 (1986).
5. Beatty, M. J., Rudd, J. E. & Valencic, K. M. A re-examination of the verbal aggressiveness scale: One factor or two? *Communication Research Reports* **16**, 10–17 (1999).
6. Lins de Holanda Coelho, G., H. P. Hanel, P. & J. Wolf, L. The Very Efficient Assessment of Need for Cognition: Developing a Six-Item Version. *Assessment* **27**, 1870–1885 (2020).
7. Kessler, S. H. & Zillich, A. F. Searching Online for Information About Vaccination: Assessing the Influence of User-Specific Cognitive Factors Using Eye-Tracking. *Health Communication* **34**, 1150–1158 (2019).
8. König, J. *et al.* Assessment of subjective emotional valence and long-lasting impact of life events: development and psychometrics of the Stralsund Life Event List (SEL). *BMC Psychiatry* **18**, 105 (2018).

9. Dricu, M. *et al.* Warmth and competence predict overoptimistic beliefs for out-group but not in-group members. *PLoS ONE* **13**, e0207670 (2018).

10. Bettencourt, B. A., Dill, K. E., Greathouse, S. A., Charlton, K. & Mulholland, A. Evaluations of Ingroup and Outgroup Members: The Role of Category-Based Expectancy Violation. *Journal of Experimental Social Psychology* **33**, 244–275 (1997).

11. Huang, J. L., Curran, P. G., Keeney, J., Poposki, E. M. & DeShon, R. P. Detecting and Deterring Insufficient Effort Responding to Surveys. *J Bus Psychol* **27**, 99–114 (2012).

12. Yuan, S., Besley, J. C. & Ma, W. Be mean or be nice? Understanding the effects of aggressive and polite communication styles in child vaccination debate. *Health Communication* **34**, 1212–1221 (2019).

13. Haghighat, R. The Development of the Brief Social Desirability Scale (BSDS). *EJOP* **3**, (2007).

14. Blanca, M. J., Alarcón, R. & Arnau, J. Non-normal data: Is ANOVA still a valid option? *Psicothema* 552–557 (2017) doi:10.7334/psicothema2016.383.
