# Supplementary material for: Hostility has a trivial effect on persuasiveness of rebutting science denialism on social media
Source: Commun Psychol. 2023 Dec 11;1:39. doi: 10.1038/s44271-023-00041-w (PMC11332242; doi:10.1038/s44271-023-00041-w)
Supplement: Supplementary file 3 — Reporting Summary [file 44271_2023_41_MOESM3_ESM.pdf]

nature portfolio

Corresponding author(s): Philipp Schmid
Last updated by author(s): Nov 8, 2023

Reporting Summary

Nature Portfolio wishes to improve the reproducibility of the work that we publish. This form provides structure for consistency and transparency in reporting. For further information on Nature Portfolio policies, see our Editorial Policies and the Editorial Policy Checklist.

Statistics

For all statistical analyses, confirm that the following items are present in the figure legend, table legend, main text, or Methods section.

n/a	Confirmed	
☐	☒	The exact sample size (*n*) for each experimental group/condition, given as a discrete number and unit of measurement
☐	☒	A statement on whether measurements were taken from distinct samples or whether the same sample was measured repeatedly
☐	☒	The statistical test(s) used AND whether they are one- or two-sided<br>*Only common tests should be described solely by name; describe more complex techniques in the Methods section.*
☐	☒	A description of all covariates tested
☐	☒	A description of any assumptions or corrections, such as tests of normality and adjustment for multiple comparisons
☐	☒	A full description of the statistical parameters including central tendency (e.g. means) or other basic estimates (e.g. regression coefficient) AND variation (e.g. standard deviation) or associated estimates of uncertainty (e.g. confidence intervals)
☐	☒	For null hypothesis testing, the test statistic (e.g. *F*, *t*, *r*) with confidence intervals, effect sizes, degrees of freedom and *P* value noted<br>*Give P values as exact values whenever suitable.*
☒	☐	For Bayesian analysis, information on the choice of priors and Markov chain Monte Carlo settings
☒	☐	For hierarchical and complex designs, identification of the appropriate level for tests and full reporting of outcomes
☐	☒	Estimates of effect sizes (e.g. Cohen's *d*, Pearson's *r*), indicating how they were calculated

Our web collection on statistics for biologists contains articles on many of the points above.

Software and code

Policy information about availability of computer code

Data collection	Data of all experiments in the study were collected using the web-based Enterprise Feedback Suite (EFS) by Tivian.
Data analysis	We used the statistical software R version 4.2.2 and the following additional packages for analyses: metafor version 3.8.1, emmeans 1.8.2, dplyr 1.0.10, pryr 0.1.5, Cairo 1.6.0, car 3.1.1, ggpubr 0.5.0, ggplot2 3.4.0, foreign 0.8.83, PROCESS for R version 4.0; We used SPSS version 28 for data provided in tables in the Supplement and for scatter plots. For equivalence testing we used the TOSTER spreadsheet.

For manuscripts utilizing custom algorithms or software that are central to the research but not yet described in published literature, software must be made available to editors and reviewers. We strongly encourage code deposition in a community repository (e.g. GitHub). See the Nature Portfolio guidelines for submitting code & software for further information.

Data

Policy information about availability of data

All manuscripts must include a data availability statement. This statement should provide the following information, where applicable:

- Accession codes, unique identifiers, or web links for publicly available datasets
- A description of any restrictions on data availability
- For clinical datasets or third party data, please ensure that the statement adheres to our policy

The datasets for all experiments and figures are available at Open Science Framework. The DOI to access the datasets is https://doi.org/10.17605/OSF.IO/HG2Y8. The permanent weblink is https://osf.io/hg2y8/.

Human research participants

Policy information about studies involving human research participants and Sex and Gender in Research.

Reporting on sex and gender	Gender was measured for sample description. Gender was determined based on self-reporting. Sex was not measured. Gender was not part of the primary study design. Gender was not part of the primary analyses as this study does not focus on gender differences and because the authors could not derive theory-based hypotheses about the role of gender in this study. Experiment 1: Gender: 47.6% women; 51.7% men; 0.6% non-binary Experiment 2: Gender: 50% women; 47.4% men; 2.6% non-binary Experiment 3: Gender: 48.8% women; 48.8% men; 2.3% non-binary Experiment 4: Gender: 46.7% women; 51.1% men, 2.1% non-binary
Population characteristics	See below
Recruitment	All experiments were conducted online using Prolific.co for recruiting. Participation was a voluntary decision and participants could quit the survey at any time. Therefore, individuals intrinsically interested in the topic of the experiments could have been more willing to finalize the study. We tried to reduce this potential bias with adequate compensation of participants.
Ethics oversight	Institutional Review Board of the University of Erfurt

Note that full information on the approval of the study protocol must also be provided in the manuscript.

Field-specific reporting

Please select the one below that is the best fit for your research. If you are not sure, read the appropriate sections before making your selection.

☐ Life sciences ☒ Behavioural & social sciences ☐ Ecological, evolutionary & environmental sciences

For a reference copy of the document with all sections, see nature.com/documents/nr-reporting-summary-flat.pdf

Behavioural & social sciences study design

All studies must disclose on these points even when the disclosure is negative.

Study description	All experiments in the study are quantitative experimental.
Research sample	Experiment 1: Prolific.co sample. 521 U.S. adults (Age: Mean = 34.05, Standard Deviation = 10.46; Gender: 47.6% women; Education: 96.7% reported having a high school diploma or higher education but no doctorate/PhD degree) Experiment 2: Prolific.co sample. 310 U.S. adults (Age: M = 34.46, SD = 11.25; Gender: 50% women; Education: 94.2% reported having a high school diploma or higher education but no doctorate/PhD degree) Experiment 3: Prolific.co sample. 1200 U.S. adults (Age: M = 33.00, SD = 12.17; Gender: 48.8% women; Education: 95.1% reported having a high school diploma or higher education but no doctorate/PhD degree) Experiment 4: Prolific.co sample. 1195 U.S. adults (Age: M = 33.71, SD = 12.12; Gender: 46.7% women; Education: 94.6% reported having a high school diploma or higher education but no doctorate/PhD degree) Reason for sample selection: U.S. online users were considered a relevant target group for the research questions because the study focuses on the impact of hostile language in online environments and hostility is frequently experienced in online discussions in the US.
Sampling strategy	Convenience sampling procedure for all experiments. For Experiment 1, we aimed for statistical power of at least .8 to detect the hypothesized main effects and pairwise comparisons in a 2x2-between-subjects-ANOVA, given α = .05 and assumed effect sizes of d ≥ 0.35 (informed by similar experiments involving civility). For Experiment 2, the targeted smallest effect size for the main effects was d = .37 based on similar experiments involving rebuttal vs. no rebuttal comparisons. In Experiments 3 and 4, we aimed to detect d ≥ .2 for the main effects as the smallest effect size of interest (SESOI). References for effect sizes are provided in preregistration protocols.
Data collection	Data of all experiments in the study were collected using the web-based Enterprise Feedback Suite (EFS) by Questback. Data was stored and analyzed on a computer. Owing to the randomization process, the investigators were blind to the group allocation process.
Timing	Experiment 1: 22.09.2020 - 16.10.2020<br>Experiment 2: 08.03.2021 - 01.04.2021<br>Experiment 3: 18.06.2021 - 01.07.2021<br>Experiment 4: 23.06.2021 - 16.07.2021
Data exclusions	All data from participants were included in the primary analysis without any exclusions. Exploratory robustness analyses excluding specific participants (e.g. speeders) are provided in the manuscript.

Non-participation	Experiment 1: N = 592 participants clicked on the link of the study, 541 proceeded after the introduction page and 521 finished the experiment.<br>Experiment 2: N = 353 participants clicked on the link of the study, 324 proceeded after the introduction page and 310 finished the experiment.<br>Experiment 3: N = 1351 clicked on the link of the study, 1249 proceeded after the introduction page and 1200 finished the experiment.<br>Experiment 4: N = 1427 clicked on the link of the study, 1277 proceeded after the introduction page and 1195 finished the experiment.<br>Reasons of individuals for not finishing the experiments are unknown.
Randomization	Participants of all experiments in the study were randomly allocated to one out of four (Experiment 1 & Experiment 2) or six (Experiment 3 & Experiment 4) experimental conditions. An automatic randomization mechanism provided by the Enterprise Feedback Suite (EFS) by Questback was used for randomization. At the start of the study, the software randomly selected which rebuttal information was to be communicated to participants.

Reporting for specific materials, systems and methods

We require information from authors about some types of materials, experimental systems and methods used in many studies. Here, indicate whether each material, system or method listed is relevant to your study. If you are not sure if a list item applies to your research, read the appropriate section before selecting a response.

Materials & experimental systems

n/a	Involved in the study	
☒	☐	Antibodies
☒	☐	Eukaryotic cell lines
☒	☐	Palaeontology and archaeology
☒	☐	Animals and other organisms
☒	☐	Clinical data
☒	☐	Dual use research of concern

Methods

n/a	Involved in the study	
☒	☐	ChIP-seq
☒	☐	Flow cytometry
☒	☐	MRI-based neuroimaging
